# Gut microbiota-mediated Gene-Environment interaction in the *TashT* mouse model of Hirschsprung disease

**DOI:** 10.1038/s41598-018-36967-z

**Published:** 2019-01-24

**Authors:** Aboubacrine Mahamane Touré, Mathieu Landry, Ouliana Souchkova, Steven W. Kembel, Nicolas Pilon

**Affiliations:** 1 Département des Sciences Biologiques, Université du Québec à Montréal, Montréal, H3C 3P8 Québec Canada; 2 Centre d’Excellence en Recherche sur les Maladies Orphelines – Fondation Courtois (CERMO-FC), Université du Québec à Montréal, Montréal, H2X 3Y7 Québec Canada; 3 Département d’Enseignement et de Recherche de Biologie de la Faculté des Sciences et Techniques de l’Université des Sciences, des Techniques et des Technologies de Bamako, Badalabougou, Colline de Badala, Bamako, Mali

## Abstract

Based on the bilateral relationship between the gut microbiota and formation/function of the enteric nervous system (ENS), we sought to determine whether antibiotics-induced dysbiosis might impact the expressivity of genetically-induced ENS abnormalities. To address this, we took advantage of the *TashT* mouse model of Hirschsprung disease, in which colonic aganglionosis and hypoganglionosis are both much more severe in males. These defects result into two male-biased colon motility phenotypes: either megacolon that is lethal around weaning age or chronic constipation in adults, the latter being also associated with an increased proportion of nitrergic neurons in the distal ENS. Induction of dysbiosis using a cocktail of broad-spectrum antibiotics specifically impacted the colonic ENS of *TashT*^Tg/Tg^ mice in a stage-dependent manner. It further decreased the neuronal density at post-weaning age and differentially modulated the otherwise increased proportion of nitrergic neurons, which appeared normalized around weaning age and further increased at post-weaning age. These changes delayed the development of megacolon around weaning age but led to premature onset of severe constipation later on. Finally, local inhibition of nitric oxide signaling improved motility and prevented death by megacolon. We thus conclude that exposure to antibiotics can negatively influence the expressivity of a genetically-induced enteric neuropathy.

## Introduction

Hirschsprung disease (HSCR) is a congenital malformation of the enteric nervous system (ENS) that globally affects 1/5000 live births, with an intriguing 4:1 male-biased sex ratio^1^. HSCR is characterized by the absence of enteric neural ganglia (aganglionosis) over a varying length of the distal colon, caused by the failure of neural crest-derived ENS progenitors to reach this region during prenatal development^2^. One of the primary roles of the ENS is to establish rhythmic patterns of contraction and relaxation in enteric smooth muscle layers, thereby controlling bowel motility. In HSCR, the aganglionic segment remains constantly contracted, leading to obstruction and distention of the proximal colon (megacolon) because of excessive accumulation of fecal material. Etiology of HSCR is considered to be multifactorial, with a genetic contribution generally believed to be heavier than that of non-genetic factors^3,4^. However, most if not all of the known HSCR-associated genetic variants are partially penetrant^5^, suggesting that many HSCR cases do have a critical non-genetic contribution^6^. This contribution would most likely only be evident in the presence of HSCR-associated genetic changes that cause aganglionosis but not over a sufficiently long segment to result in functional obstruction. In these circumstances, even a small contribution of non-genetic factors to the extent of aganglionosis would render functional obstruction inevitable.

The prenatal period, during which the developing intestines are being colonized by neural crest-derived ENS progenitors, is presumably highly vulnerable to non-genetic factors. In support of this idea, vitamin A deficiency or exposure to drugs like ibuprofen and mycophenolate mofetil during intrauterine development were all found to increase the extent of aganglionosis in HSCR mouse models^7–9^. Yet, the contribution of non-genetic factors to HSCR is not expected to be restricted to prenatal development. The postnatal maturation of the ENS, which lasts several weeks after birth^10^, is most likely another sensitive period. A tempting hypothesis would be that non-genetic factors might increase HSCR risk by worsening the ENS defects typically observed in the transition zone just upstream of the aganglionic segment, such as decreased neuronal density (hypoganglionosis) and neuronal subtype imbalance^11–15^. This would provide a plausible explanation as to why the age at which clinical symptoms of HSCR manifest varies across affected children^1^. However, this possibility has not been tested yet.

With an overall genetic composition much larger than the genome of their host^16^, the gut microbiota stands out as a good candidate for postnatally influencing HSCR incidence. This complex microbial community is established through a colonization phase, commonly believed to occur at birth, followed by a maturation phase that is intertwined with the postnatal maturation of the ENS^17^. Accordingly, depletion and/or perturbation of the gut microbiota during early postnatal stages in mice have been linked to alteration of ENS structure and bowel motility^18,19^. At least part of these defects are apparently due to dysregulated activation of Toll-like receptors (TLRs) by microbial products at the surface of enteric neurons and glial cells^20–23^. It is especially striking that the ENS defects observed upon perturbation of the gut microbiota or TLR signaling are very similar to those observed in the transition zone of HSCR mouse models, being predominantly characterized by decreased neuronal density, neuronal subtype imbalance (mostly affecting nitrergic neurons) and/or abnormal development of glial cells^18,19,22–24^.

The *TashT* mouse line is ideally suited for studying gene-environment interactions in the context of HSCR. This line was generated via an insertional mutation screen for loci with key roles in neural crest cells, which for *TashT* consists of a silencer-enriched region in a chromosome 10 gene desert^25^. The transgenic insertion in this region directly perturbs the expression of *Fam162b*^26^ as well as other genes to be described elsewhere. The *TashT* line is unique in that it is the only HSCR mouse model that recapitulates both the partial penetrance and the male bias of the human condition^26^. Both of these characteristics can be explained by the fact that the extent of aganglionosis in homozygous *TashT* animals (*TashT*^Tg/Tg^) is close to a “tipping point” for developing functional obstruction, with females generally having a shorter aganglionic segment^26^. In the end, megacolon is observed almost exclusively in male pups, causing the death of about a quarter of them around weaning age (postnatal day (P) 21)^26^. Otherwise, *TashT*^Tg/Tg^ mice are able to evacuate fecal material and survive when more than 4/5 of their colon is innervated. However, we found that surviving males, but not females, suffer from chronic constipation after reaching adulthood (2–3 months old)^15^. This phenotype is associated with ENS defects in the transition zone that are either more severe (hypoganglionosis) or specifically observed (increased proportion of nitrergic neurons) in males^15^.

Studies aimed at analyzing perturbations of the gut microbiota in the context of HSCR have so far been focused on the risk of developing HSCR-associated enterocolitis (HAEC)^27–29^. In the current study, we used the *TashT* line in order to test the different hypothesis that antibiotics-induced perturbations of the microbial flora – that we term “dysbiosis” in the current context – during the early postnatal period might increase the incidence of aganglionic megacolon. Intriguingly, we found that the global incidence of megacolon is not changed in immature *TashT*^Tg/Tg^ male mice exposed to antibiotics, but that the time window over which this phenotype is more likely to manifest in normal conditions is shifted by several days in antibiotics-treated *TashT*^Tg/Tg^ male mice. This is mainly associated with dysbiosis-associated temporal modulation of the proportion of nitrergic neurons in the transition zone, which is decreased at P22–23 and increased at P30–36. We further discovered that local inhibition of nitric oxide (NO) signaling using rectal enemas containing NG-nitro-L-arginine methyl ester (L-NAME) may have therapeutic value for colonic dysmotility. Our work thus strongly suggests that antibiotics-induced dysbiosis might contribute to late-onset HSCR risk, highlighting a key pathogenic role for the transition zone.

## Results

### Taxonomic composition of the colonic microbiota in wild-type, *TashT*^Tg/Tg^ and *Holstein*^Tg/Tg^ mice

**Figure 1:**
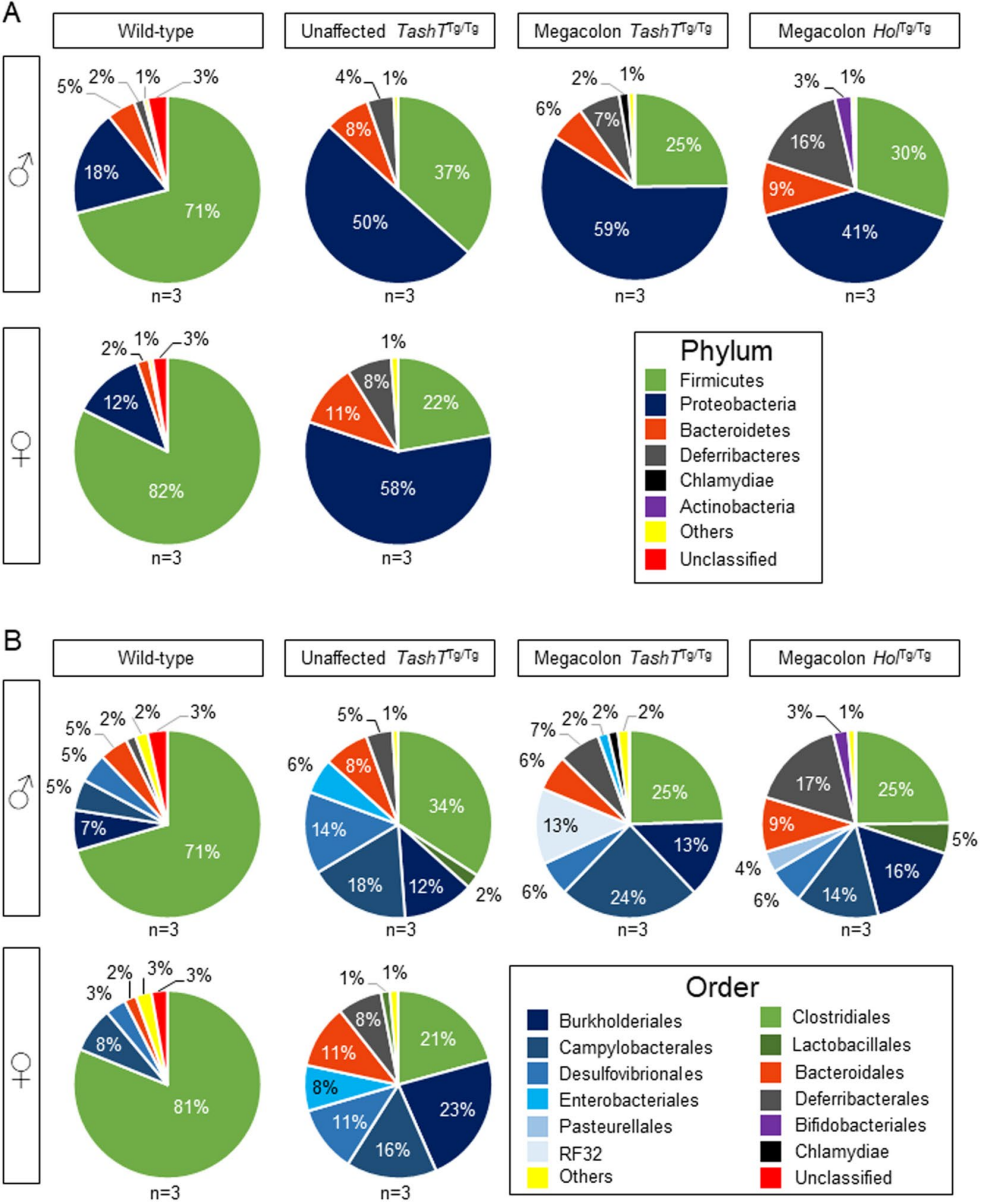
Basal bacterial composition of colonic content from wild-type, *TashT*^Tg/Tg^ (with or without megacolon) and *Holstein*^Tg/Tg^ mice at P21–22. (**A**,**B**) The pie charts display the relative average abundance of *16S* rRNA gene sequences at the phylum (**A**) and order (**B**) levels. Taxonomic groups are color-coded and indicated on the bottom right side of each panel. “Others” and “Unclassified” stand for the sum of low-abundance (<1.5%) taxa or yet unknown taxa, respectively.

To determine how the *TashT* line is comparable to other HSCR mouse models in terms of microbiota composition, we first profiled the basal microbiome in the colon of *TashT*^Tg/Tg^ pups in comparison to wild-type (WT) animals of weaning age (P21–22). Given the partial penetrance and male bias of the megacolon phenotype in this mutant line^26^, we also took care to perform this analysis as a function of disease state (with or without megacolon) and sex. Moreover, to determine whether a general microbiome signature might be associated with megacolon, we included another of our previously described HSCR mouse models called *Holstein* (involving overproduction of Collagen VI)^30^. Using targeted sequencing of the *16S* rRNA gene to profile the microbiome, we first noted a comparable signature in WT mice of both sexes, this signature consisting of the phyla *Firmicutes*, *Proteobacteria* and *Bacteroidetes* (in order of abundance) (Figs 1A and S1A). Microbiome profiling of *TashT*^Tg/Tg^ and *Holstein*^Tg/Tg^ mutant mice revealed a clear dysbiotic state in both cases, reminiscent of the previously reported dysbiosis in the *Ednrb*-null HSCR mouse model^28,29^. The observed dysbiosis was largely similar in both of our mutant lines (regardless of sex and health status), being mainly characterized by a marked decrease in abundance of *Firmicutes*, a marked increase of *Proteobacteria*, and a variable increase of *Deferribacteres* (Figs 1A, S2A, S3A and S4). At the order level, a small difference (<10% of all taxa) was noted as a function of sex, with *Burkholderiales* being only detected in WT males (Figs 1B and S1B). Small changes (<15% of all taxa) were also noted between mutant lines as a function of both disease state and genotype. Megacolon-suffering *TashT*^Tg/Tg^ males differed from *TashT*^Tg/Tg^ males unaffected by megacolon by the emergence of *RF32*, whereas *Bifidobacteriales* and *Pasteurellales* were exclusively detected in *Holstein*^Tg/Tg^ mice (Figs 1B, S2B and S3B). Given that the differences in microbiota composition appear minor between mutants in terms of relative abundance, our observations suggest the existence of a general microbiome signature in HSCR mouse models. However, the fact that a broadly similar dysbiotic state is present in all *TashT*^Tg/Tg^ animals, regardless of the presence of megacolon (Figs S2 and S4), indicates that microbiota alterations are mainly caused by ENS defects and not by bowel obstruction. Moreover, although critical sex-based differences in gut microbiota have been previously reported in non-obese diabetic (NOD) mice^31^, our data suggest that sex-based differences are subtle in the context of HSCR (Fig. S3).

### Impact of early continuous antibiotic treatment on the fecal microbiota of wild-type and *TashT*^Tg/Tg^ male pups

**Figure 2:**
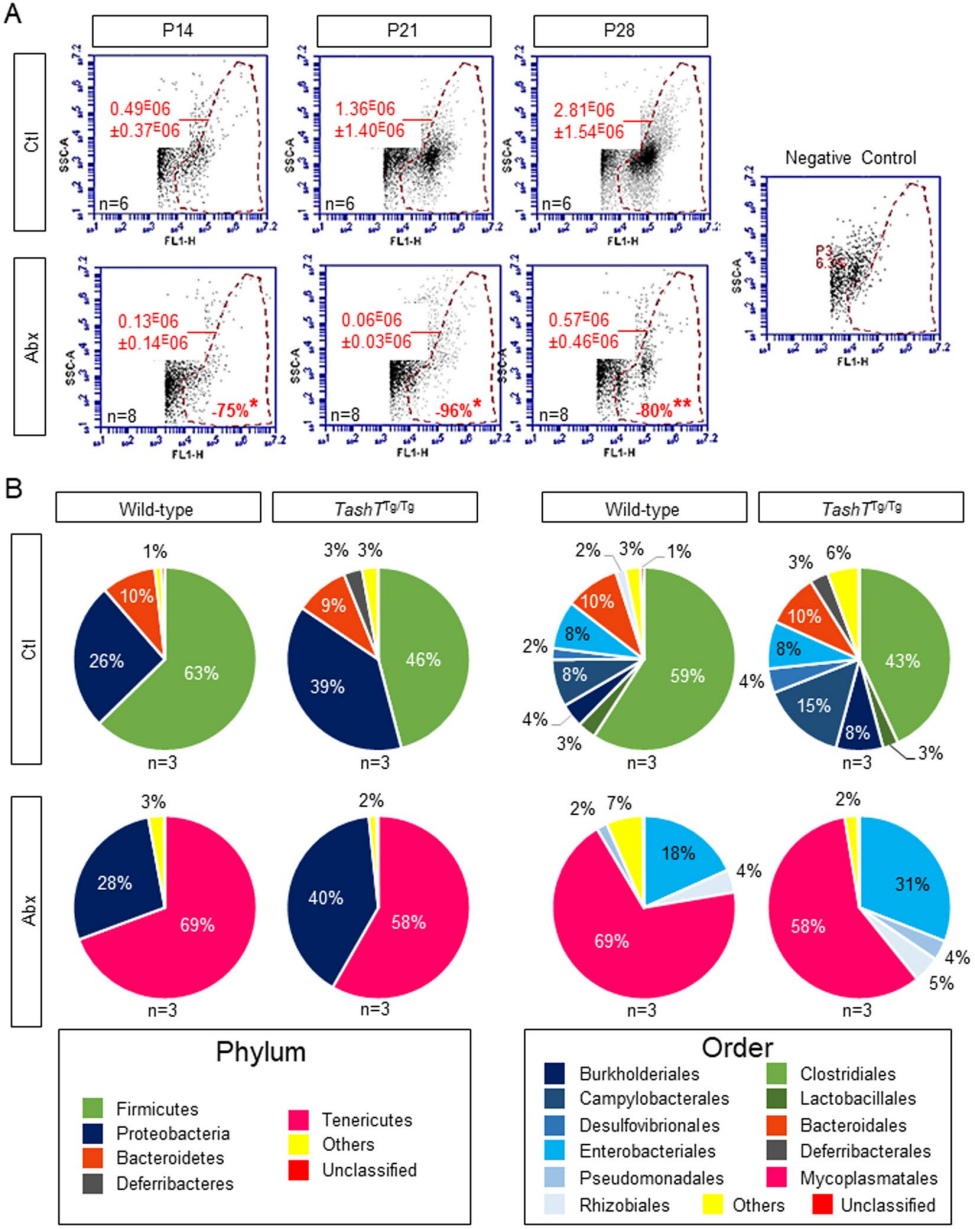
Antibiotics-induced dysbiosis in wild-type and *TashT*^Tg/Tg^ mice at P28–30. (**A**) Flow cytometry-based quantitative analysis of SYTO 13-labeled bacteria in stool homogenates prepared from antibiotics-treated (Abx) mice in comparison to untreated (Ctl) mice at P14, P21 and P28. At each stage, antibiotics-induced reduction of the average concentration of bacteria per µl of stool homogenate (red numbers on the left of each panel) was determined and expressed as percentage difference (red numbers at the bottom of lower panels) of the average bacterial concentration in untreated mice. A blank solution without bacteria (negative control) was used for gating purposes. (**B**) Profile of bacteria extracted from stool pellets of P28–30 WT and *TashT*^Tg/Tg^ males exposed or not to antibiotics in their drinking water, which was sweetened with sucrose. The pie charts display the relative average abundance of *16S* rRNA gene sequences at the phylum and order levels. Taxonomic groups are color-coded and indicated at the bottom of each panel. “Others” and “Unclassified” stand for the sum of low-abundance (<1.5%) taxa or yet unknown taxa, respectively. (*P < 0.05, **P < 0.01; Student’s *t*-test).

To induce gut dysbiosis during the early postnatal period, mouse pups and their mother were continuously exposed to a cocktail of antibiotics added to drinking water^24^, starting before birth until P36. Efficiency of this approach was validated by evaluating the concentration of fecal bacteria at P14, P21 and P28, which revealed marked decreases of 75%, 96% and 80%, respectively (Fig. 2A). The impact of antibiotic treatment on microbiota composition was then assessed by profiling the fecal microbiome from WT and *TashT*^Tg/Tg^ mice at P28–30, again via targeted sequencing of the *16S* rRNA gene. Of note, for this analysis and the remaining of the current study, only male mice were evaluated because of the strong male bias of *TashT*^Tg/Tg^ ENS defects^15,26^. In absence of antibiotics, the changes in microbiota composition previously observed between WT and *TashT*^Tg/Tg^ mice at P21–22 (Fig. 1A,B) appeared to be maintained at P28–30, at both the phylum and the order levels (Fig. 2B). Interestingly, despite this difference, the impact of antibiotics was found to be very similar in both WT and *TashT*^Tg/Tg^ mice (Figs 2B and S5). At the phylum level, exposure to antibiotics resulted in the marked emergence of *Tenericutes* at the expense of *Firmicutes* and *Bacteroidetes* (Figs 2B and S6A). At the order level, these changes were mainly characterized by the marked emergence of *Mycoplasmatales* and an increase of *Enterobacteriales*, at the expense of *Clostridiales*, *Bacteroidales*, *Campylobacterales* and *Burkholderiales* (Figs 2B and S6B).

### Impact of antibiotics-induced dysbiosis on motility parameters in *TashT*^Tg/Tg^ male mice

**Figure 3:**
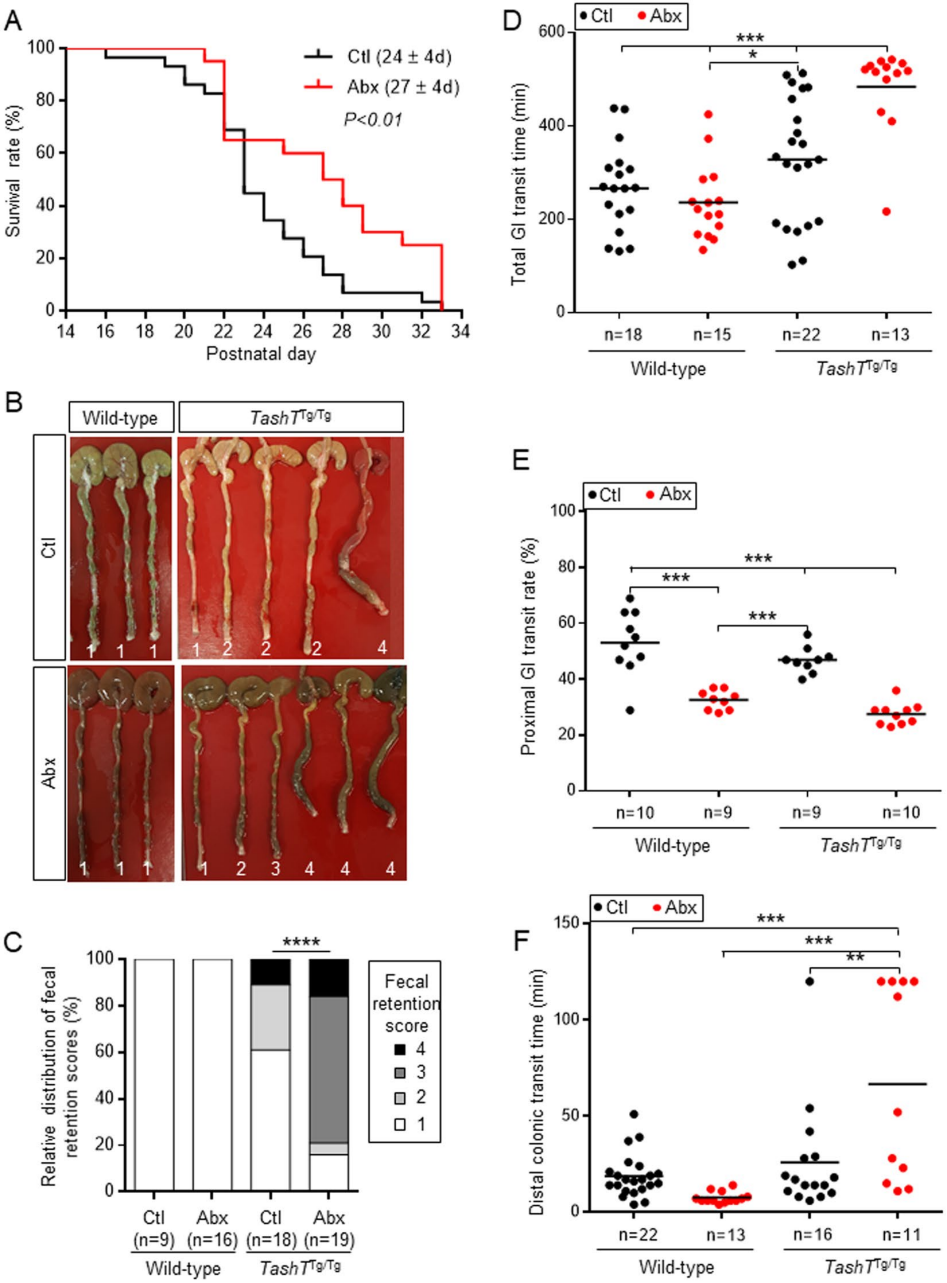
Antibiotics-induced dysbiosis specifically impacts survival and motility parameters of *TashT*^Tg/Tg^ male mice. (**A**) Kaplan-Meier curve showing the age of death of megacolon-suffering *TashT*^Tg/Tg^ male mice exposed (Abx) or not (Ctl) to antibiotics (n = 29 for Ctl group and 20 for Abx group). Average age of death is indicated between parentheses. (**B**) Representative images of whole colons from P33–36 WT or *TashT*^Tg/Tg^ male mice treated (Abx) or not (Ctl) with antibiotics. Numbers are arbitrary units assigned by someone who was blinded to experimental conditions and genotypes in order to grade fecal retention severity: 1-Well-separated pellets as observed in the colon of WT mice; 2-Presence of compacted feces of any consistence restricted to the most distal third of the colon; 3-Presence of compacted feces of any consistence in the most distal third of the colon and beyond; 4-Megacolon-like phenotype characterized by distal obstruction and proximal distention. (**C**) Relative distribution of fecal retention scores within each mouse group, using the arbitrary units described in B. (**D**–**F**) GI motility parameters in WT and *TashT*^Tg/Tg^ male mice exposed (Abx) or not (Ctl) to antibiotics. Total GI transit (**D**) was evaluated by the time (minutes) required for expelling the first red pellet following oral gavage with carmine red dye. Of note, mice for which this amount of time was ≥480 minutes (8 hours) are those that did not expel red pellets at the end of the manipulation. Proximal GI transit (**E**) was evaluated by the distance travelled by the carmine red dye 15 min after gavage and expressed in percentage of the total length of the small intestine. Distal colonic transit (**F**) was evaluated using the bead latency assay and expressed in time (minutes) required to expel the bead. Distal colonic transit time was capped to 120 minutes (2 hours) in order to simplify the graph without altering statistical significances. (*P < 0.05, **P < 0.01, ***P < 0.001, ****P < 0.0001; Mantel-Cox test in **A**, Chi-square test in **C**, and one-way ANOVA with Tukey’s post hoc test in **D**–**F**).

Having validated that our antibiotic treatment led to severe dysbiosis, we next sought to determine whether this approach could exacerbate transition zone-associated ENS defects in *TashT*^Tg/Tg^ male mice. In such case, antibiotics-induced dysbiosis would be expected to increase the incidence of either megacolon or chronic constipation, as both of these motility-related phenotypes have been previously reported in *TashT*^Tg/Tg^ male mice^15,26^. Close follow-up of a large number of *TashT*^Tg/Tg^ pups first revealed that death by megacolon did not occur more frequently upon antibiotic treatment, being observed in 18.95% of untreated (29 out of 153 animals) and 18.87% of antibiotics-treated (20 out of 106 animals) males. Intriguingly, we instead observed that exposure to antibiotics delayed age of death by 3 days on average (Fig. 3A). Visual inspection of colons during the last days of treatment (P33–36) further revealed that surviving *TashT*^Tg/Tg^ males that do not exhibit any of the clinical signs of megacolon in rodents (i.e. lethargy, hunched posture, diarrhea and/or distended abdomen) did exhibit a marked increase of fecal retention upon antibiotic treatment (Fig. 3B). Quantification of fecal retention using arbitrary units revealed that both the incidence and the severity of this phenotype were affected (Fig. 3C). Moreover, such effect appeared specific to *TashT*^Tg/Tg^ males, as no fecal retention was observed in the colon of antibiotics-treated WT animals (Fig. 3B,C). Yet, when compared to untreated animals, all antibiotics-treated mice displayed the expected phenotypic signature^24,32^, which include growth retardation (Fig. S7A), presence of a brown (Fig. 3B) and enlarged (Fig. S7B) cecum, and depletion of mucosal glial cells (Fig. S7C).

To complement the morphological analyses described above, we then directly assessed the transit of luminal content throughout the bowel in another group of P30–36 male mice. Using carmine red dye as tracer, we found that the antibiotic treatment specifically delayed the total gastro-intestinal (GI) transit time in *TashT*^Tg/Tg^ males (Fig. 3D), whereas it decreased the transit rate in the small intestine of both controls and mutants (Fig. 3E). In the colon, glass bead expulsion assays revealed that the antibiotic treatment impacted transit time in opposite directions depending of mouse genotype. Indeed, while exposure to antibiotics accelerated the average transit time by 2.5-fold (from 18.8 ± 2.4 min to 7.5 ± 0.8 min) in WT males, the same treatment delayed the average transit time by 2.6-fold (from 25.8 ± 7.0 min to 66.6 ± 15.3 min) in *TashT*^Tg/Tg^ males (Fig. 3F). These data thus suggest that the *TashT*^Tg/Tg^-specific delay of total GI transit time in response to antibiotic treatment is due to impaired transit in both the small intestine and the colon. On the other hand, the apparently normal total GI transit time in antibiotics-treated WT males can be explained by compensation of the impaired transit in the small intestine by accelerated transit in the colon.

Therefore, all of our observations strongly suggest that antibiotics-induced dysbiosis accelerates the development of male-specific chronic constipation in surviving *TashT*^Tg/Tg^ animals, a phenotype that is otherwise only evident in adults^15^. Yet, our data also indicate that antibiotics-induced dysbiosis may have a positive effect around weaning age, by somehow delaying the development of “typical” megacolon with all of the associated hallmarks including premature death.

### Impact of antibiotics-induced dysbiosis on the density and composition of the myenteric plexus in *TashT*^Tg/Tg^ male pups

In an effort to understand how antibiotics-induced dysbiosis specifically impacts colonic motility in *TashT*^Tg/Tg^ males, we examined the myenteric plexus at P22–23 (i.e. the age at which death by megacolon mostly occurs in untreated *TashT*^Tg/Tg^ males) and at P30–36 (i.e. the age at which premature onset of chronic constipation was detected in *TashT*^Tg/Tg^ males). For each time point, we analyzed the mid-distal colon (mid-DC) region expected to correspond to the intermediate zone and two control regions (duodenum and ileum), focusing on parameters that were previously shown to be affected by the *TashT* mutation (i.e. decreased neuronal density and neuronal subtype imbalance characterized by an increased proportion of nitrergic neurons)^15,26^.

**Figure 4:**
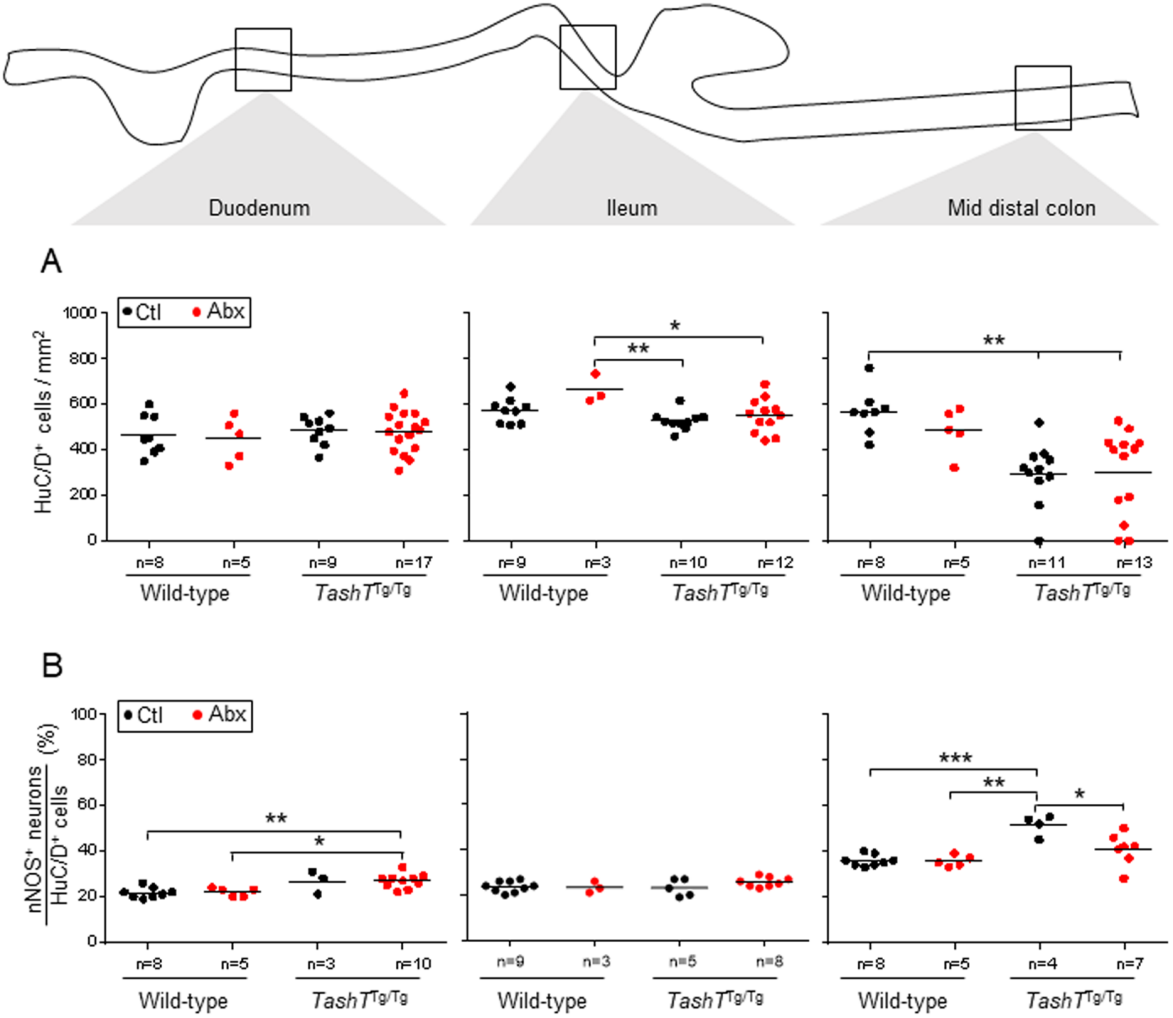
Impact of antibiotics-induced dysbiosis on neuronal density and proportion of nitrergic neurons in the myenteric plexus of wild-type and *TashT*^Tg/Tg^ male mice at P22–23. (**A**) Neuronal density was evaluated via immunofluorescence labeling of the pan-neuronal marker HuC/D and is expressed as the number of neurons per mm^2^ of longitudinal surface area. (**B**) The proportion of nitrergic (nNOS^+^) neurons was evaluated by double-immunofluorescence labeling and is expressed as the percentage of nitrergic neurons among the total number of neurons. (*P < 0.05, **P < 0.01, ***P < 0.001; one-way ANOVA with Tukey’s post hoc test).

**Figure 5:**
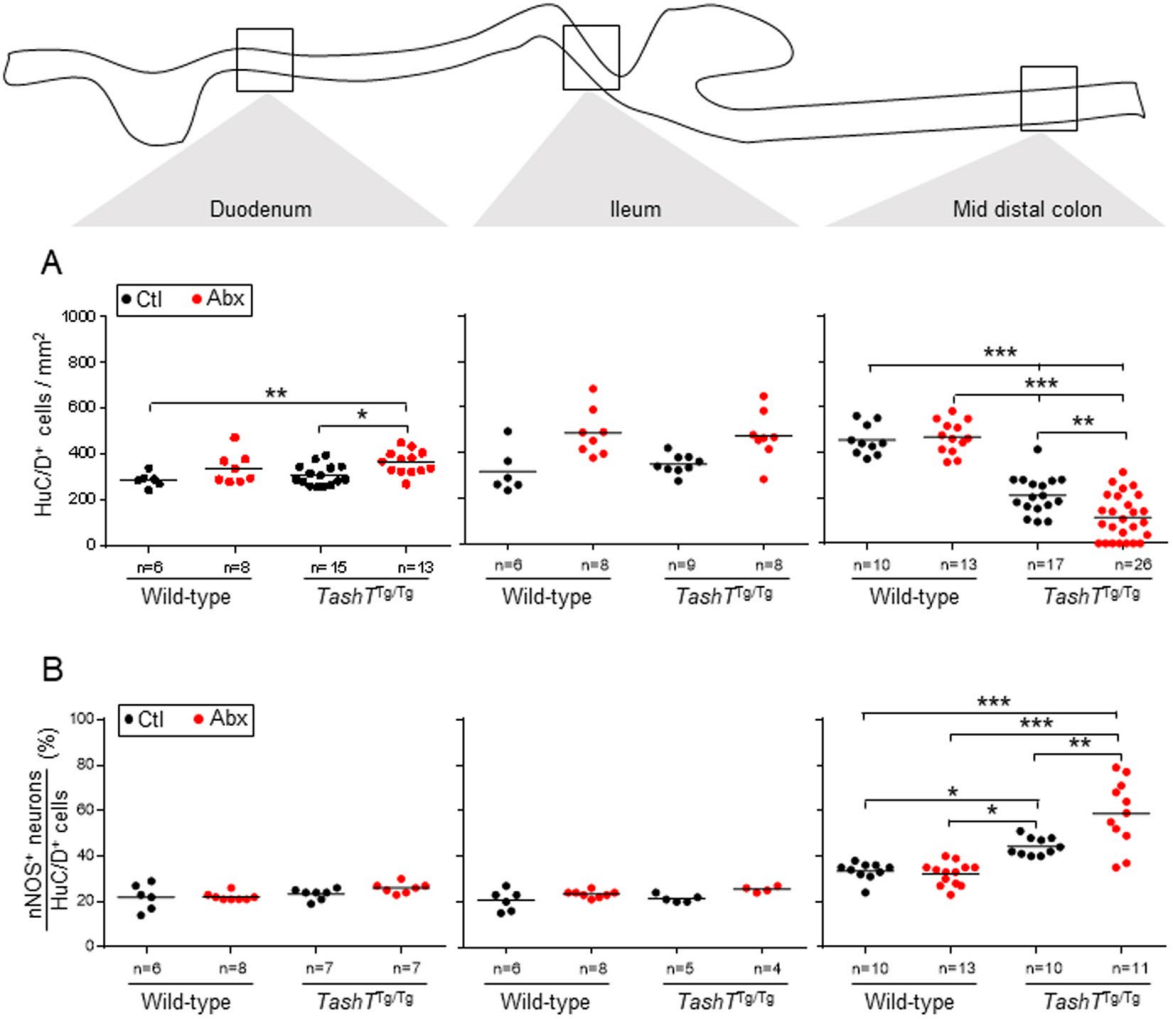
Impact of antibiotics-induced dysbiosis on neuronal density and proportion of nitrergic neurons in the myenteric plexus of wild-type and *TashT*^Tg/Tg^ male mice at P30–36. (**A**) Neuronal density was evaluated via immunofluorescence labeling of the pan-neuronal marker HuC/D and is expressed as the number of neurons per mm^2^ of longitudinal surface area. (**B**) The proportion of nitrergic (nNOS^+^) neurons was evaluated by double-immunofluorescence labeling and is expressed as the percentage of nitrergic neurons among the total number of neurons. (*P < 0.05, **P < 0.01, ***P < 0.001; one-way ANOVA with Tukey’s post hoc test).

**Figure 6:**
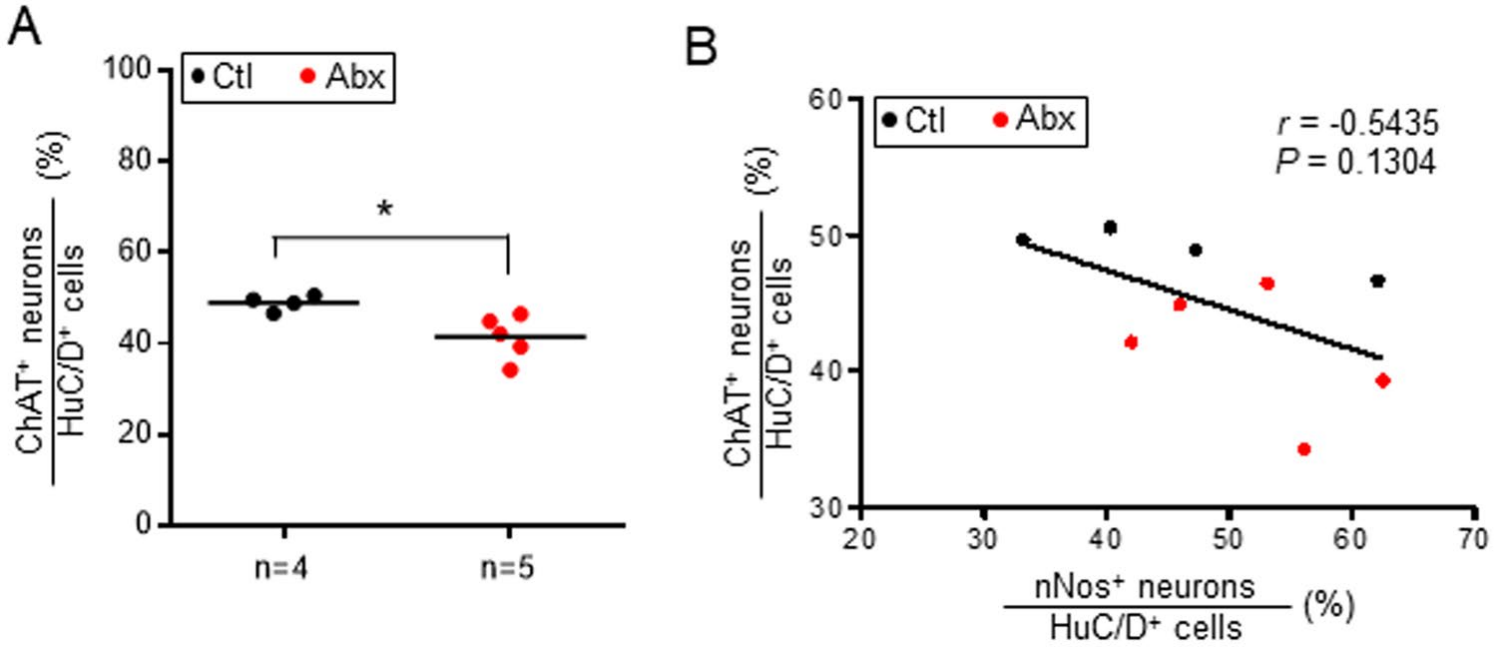
Impact of antibiotics-induced dysbiosis on the proportion of cholinergic neurons in the myenteric plexus of *TashT*^Tg/Tg^ male mice at P30–36. (**A**) The proportion of cholinergic (ChAT^+^) neurons was evaluated in the proximal-DC by double-immunofluorescence labeling and is expressed as the percentage of cholinergic neurons among the total number of neurons. (**B**) Correlation between the proportion of cholinergic neurons in the proximal-DC and the proportion of nitrergic neurons in the adjacent mid-DC. (*P < 0.05; Student’s *t*-test).

Consistent with our previous observations, untreated *TashT*^Tg/Tg^ males display a decreased neuronal density (by about 50% on average) and an increased proportion of nitrergic neurons (by about 35% on average) in comparison to untreated controls (Figs 4, 5 and S8A,B). These defects were observed at P22–23 (Fig. 4) and P30–36 (Fig. 5), being restricted to the mid-DC region. Interestingly, both of these defects were also found to be specifically modulated by antibiotics-induced dysbiosis. While neuronal density in the small intestine of P30–36 mice tends to be increased in a genotype-independent manner upon antibiotic treatment (Fig. 5A), the converse was observed in the mid-DC of *TashT*^Tg/Tg^ males, resulting in a further 50% decrease (Figs 5A and S8A) that seems to spread over the whole colon (Fig. S9A). Intriguingly, a biphasic response was observed for the proportion of nitrergic neurons, with the antibiotic treatment leading to either a decrease back to normal levels at P22–23 (Fig. 4B) or a further 34% increase at P30–36 (Figs 5B and S8B). As observed for the neuronal density, this *TashT*^Tg/Tg^-specific supplemental increase of nitrergic neurons upon antibiotic treatment appears not to be restricted to the most distal innervated segment but instead seems to propagate over the whole colon (Fig. S9B). Importantly, we further discovered that the increased proportion of nitrergic neurons in the mid-DC appears to be accompanied by a decreased proportion of cholinergic neurons in the adjacent proximal-DC (Figs 6A and S8C). However, we also found that the proportion of cholinergic neurons is not robustly correlated with the proportion of nitrergic neurons in the same animal (Fig. 6B), suggesting that other neuronal subtypes might also be affected. That being said, other key parameters of the myenteric plexus previously reported not to be affected by the *TashT* mutation (i.e. the proportion of Calretinin^+^ neurons, the neuron-glia ratio and the density of interstitial cells of Cajal) were all found to remain unaffected by the antibiotic treatment (Fig. S10).

**Figure 7:**
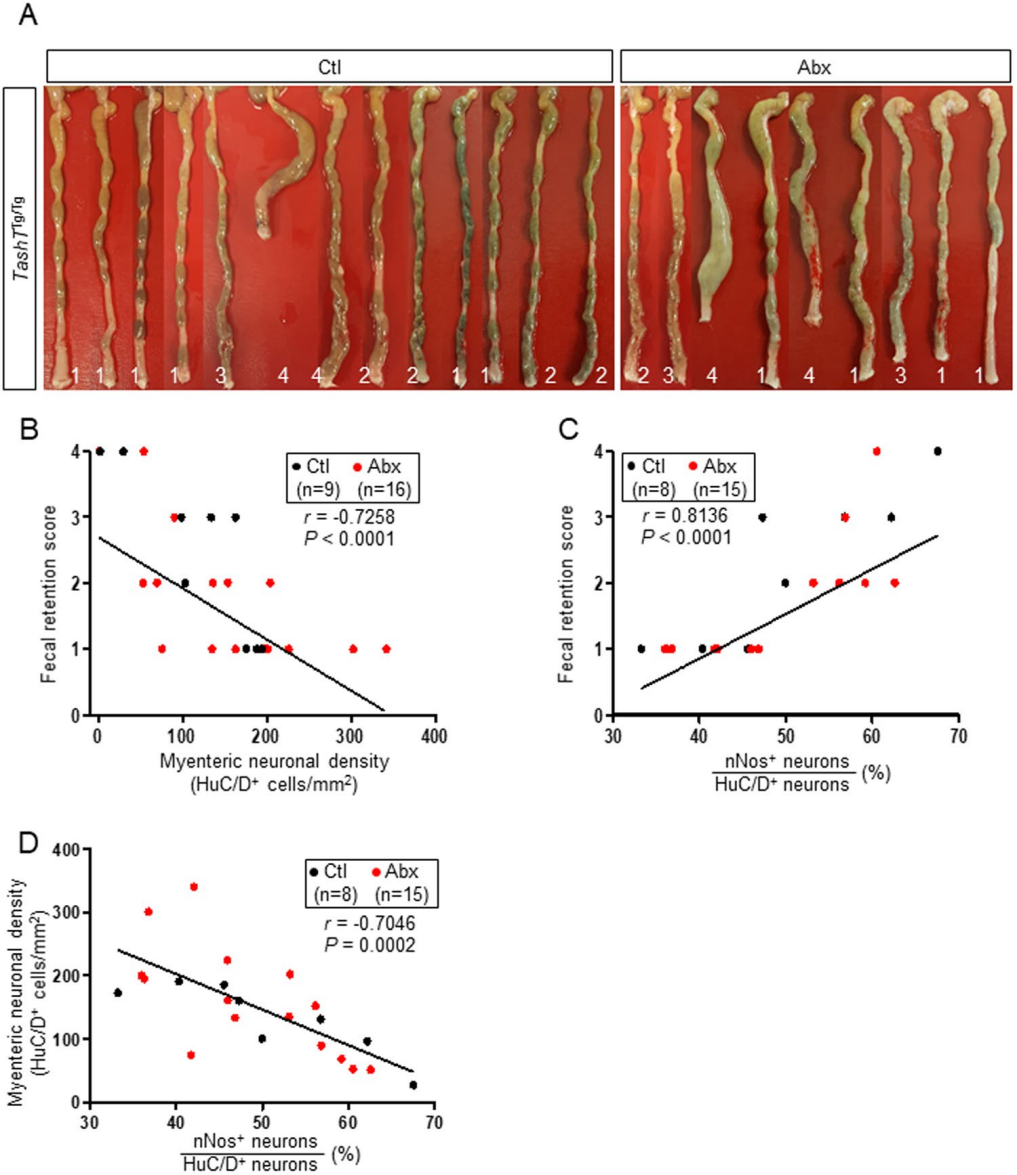
Correlations between neuronal density, increased proportion of nitrergic neurons and fecal retention in *TashT*^Tg/Tg^ male mice at P30–36. (**A**) Picture of all *TashT*^Tg/Tg^ colons used for correlation analyses in (**B**–**D**), along with their respective fecal retention score (numbers at the bottom). For each group, samples were randomly selected to ensure that the full spectrum of fecal retention scores was covered. (**B**) Correlation between fecal retention and neuronal density in the myenteric plexus of mid-DC. (**C**) Correlation between fecal retention and proportion of nitrergic neurons in the myenteric plexus of mid-DC. (**D**) Correlation between neuronal density and proportion of nitrergic neurons in the myenteric plexus of mid-DC.

Without excluding the possibility that other unknown contributing factors might also be involved, our results thus suggest that exacerbation of both of the previously reported ENS defects in adult *TashT*^Tg/Tg^ males (i.e. decreased neuronal density and increased proportion of nitrergic neurons) could be responsible for  the premature onset of chronic constipation. In support of this idea, it is also noteworthy that robust correlations were detected between both parameters and fecal retention, regardless of experimental conditions (Fig. 7). Moreover, the apparent normalization of the proportion of nitrergic neurons at P22–23 while neuronal density remains low suggests a role for this specific neuronal subtype in preventing the onset of typical megacolon.

### Impact of NO signaling inhibition on dysbiosis-induced fecal retention and life expectancy of *TashT*^Tg/Tg^ male mice

**Figure 8:**
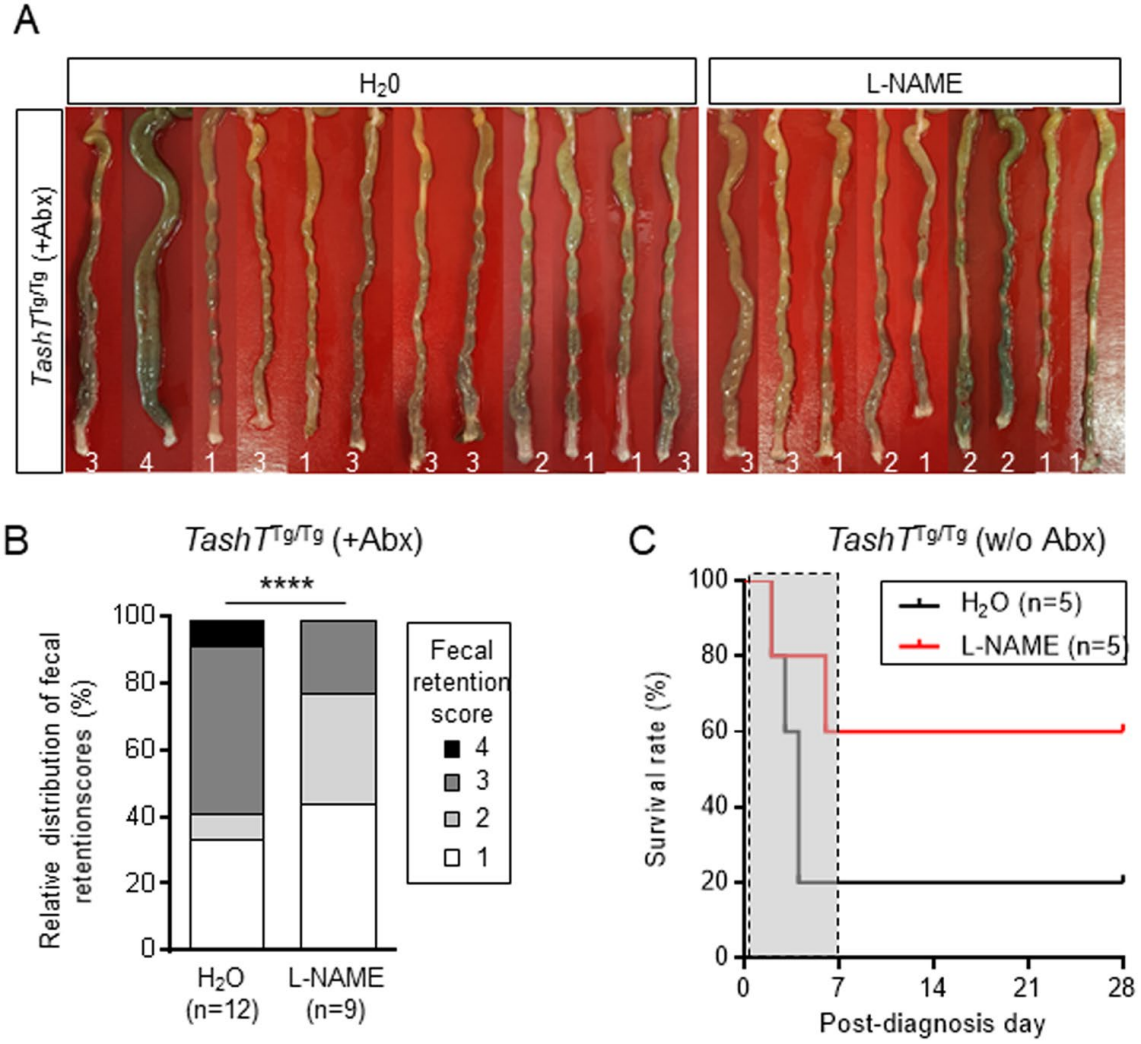
Impact of inhibition of NO signaling on fecal retention and life expectancy of *TashT*^Tg/Tg^ male mice exposed or not to antibiotics. (**A**) Picture of all antibiotics-treated *TashT*^Tg/Tg^ colons used for the quantitative analysis in B, along with their respective fecal retention score (numbers at the bottom). (**B**) Quantitative analyses of fecal retention (using the arbitrary units described in Fig. 3B,C) in P33 *TashT*^Tg/Tg^ male mice exposed to antibiotics and subjected to daily rectal enemas with L-NAME or vehicle (H_2_O) for 7 days starting at P26. (**C**) Kaplan-Meier curve showing the survival rate of megacolon-suffering P20–29 *TashT*^Tg/Tg^ male mice not exposed to antibiotics but subjected to daily rectal enemas with L-NAME or vehicle (H_2_O) for a maximum of 7 days (delineated by the grey box) starting on the day where megacolon symptoms were detected. Animal survival was then evaluated over another 3-week period, for a maximum total of 28 days post-diagnosis. (****P < 0.0001; Chi-square test).

To determine the importance of the specific increased proportion of nitrergic neurons relative to any other potential contributing factors in the dysbiosis-induced premature onset of chronic constipation, we locally reduced colonic NO signaling in antibiotic-treated *TashT*^Tg/Tg^ males by administering L-NAME through rectal enemas for 7 days starting at P26. Interestingly, we found that exposure to this widely used nitric oxide synthase (NOS) inhibitor had a tendency to attenuate the severity of fecal retention in the colon of P33 animals in comparison to control water enemas (Fig. 8A,B). Based on this observation and the fact that the proportion of nitrergic neurons is also increased in P22–23 *TashT*^Tg/Tg^ male mice not exposed to antibiotics (Fig. 4B), we next asked whether L-NAME might have therapeutic value for megacolon under normal conditions as well. To answer this question, enemas containing either L-NAME or vehicle only were administered to *TashT*^Tg/Tg^ male pups for a maximum of 7 days, starting as soon as symptoms of megacolon were detected from P20 onwards. These symptoms included growth delay, general weakness and a hunched posture. The impact of L-NAME administration on survival of megacolon-suffering *TashT*^Tg/Tg^ mice was then evaluated for a maximum of 3 weeks post-treatment. Remarkably, L-NAME appeared more potent than water at preventing death of *TashT*^Tg/Tg^ mice under these experimental conditions (Fig. 8C). Although these results should be interpreted with caution because of small sample size, our data collectively suggest that the increased proportion of nitrergic neurons play an important role in the colonic dysmotility of *TashT*^Tg/Tg^ mice regardless of the presence or the absence of antibiotic treatment.

## Discussion

Taking advantage of a unique mouse model that displays either male-biased megacolon at weaning age^26^ or male-specific chronic constipation in surviving adults^15^, we show here that antibiotics-induced dysbiosis can influence the severity of two genetically-induced ENS defects (i.e. decreased neuronal density and neuronal subtype imbalance notably characterized by an increased proportion of nitrergic neurons). The lack of impact on the global incidence of megacolon in *TashT*^Tg/Tg^ males appears to be due to a somehow protective effect around weaning age that is counterbalanced by premature onset of severe constipation later on. We observed that dysbiosis leads to a stage-dependent (post-weaning) aggravation of reduced neuronal density in the colon and a biphasic modulation of the increased proportion of nitrergic neurons, which is normalized around weaning age and then further increased later on. Importantly, all of these effects were found to be specific to *TashT*^Tg/Tg^ animals, being undetectable in WT mice. Moreover, we discovered that a local decrease of NO signaling in *TashT*^Tg/Tg^ animals can improve colonic motility and prevent death by megacolon, thereby suggesting that the nature of the transition zone is an important contributor to HSCR pathogenesis.

Besides our main objective of evaluating the impact of antibiotics-induced dysbiosis on megacolon incidence, we also wanted to determine whether *TashT*^Tg/Tg^ animals are comparable to other HSCR mouse models in terms of basal microbiota composition. Our data revealed a large degree of overlap between the microbiome profiles of *TashT*^Tg/Tg^ and *Holstein*^Tg/Tg^ mice, with both profiles significantly differing from the WT microbiome profile. At the phylum level, this dysbiotic profile was mainly characterized by a robust increase of *Proteobacteria* at the expense of *Firmicutes*. Given that a similar taxonomic profile was previously reported in a third genetically distinct HSCR mouse model (*Ednrb*-null)^28,29^, it is tempting to speculate that this particular profile might form the basis of a common microbiome signature for HSCR. Testing this possibility in human patients will clearly be challenging since information about the gut microbiota aside from HAEC is currently very limited in the context of HSCR^33^. Moreover, many HSCR patients must take antibiotics in order to prevent or treat HAEC, and antibiotic treatments can alter microbiota composition in the exact same way that is observed in HSCR mouse models^34^.

Interestingly, the dysbiotic signature described above was also noted in *TashT*^Tg/Tg^ mice without megacolon, indicating that bowel obstruction has a very limited impact on causing dysbiosis in comparison to pre-existing ENS defects. Further highlighting a prominent role for ENS defects in this regard, a similar dysbiotic signature was even observed in mice with conditional inactivation of *choline acetyltransferase* (*ChAT*) in the ENS, independently of altered transit^35^. Therefore, our findings not only strengthen the notion that the ENS is key for sculpting the microbiota^36^, but also indicate that common changes to the gut microbiota can be induced by ENS defects of different genetic origin.

In addition to serve as control group in our study, antibiotic-treated WT animals also allowed us to evaluate the impact of prolonged antibiotic treatments on the normally developing ENS. Surprisingly, although antibiotics are widely prescribed in the pediatric human population, information about their impact on ENS architecture and associated bowel motility parameters is very scarce. While the impact of a complete absence of gut microbiota (such as in germ-free mice) on early postnatal development of the ENS has been documented^19^, our study is the first – to the best of our knowledge – to similarly evaluate the impact of antibiotics-induced dysbiosis on the early postnatal ENS. Others recently addressed this question using a 2-week course of antibiotics on older WT juvenile mice (3 ± 1 weeks of age at beginning of treatment), focusing their analysis on the ileum^18^. This work notably revealed delayed proximal GI transit as well as changes in the ileal ENS characterized by a decrease of neuronal density accompanied by neuronal subtype imbalance consisting of a slightly decreased proportion of neuronal (n)NOS^+^ neurons and an increased proportion of substance P^+^ neurons^18^. While our proximal GI motility data (Fig. 3E) are in agreement with this study, this is not the case for the associated ENS defects as we instead observed a slight increase of neuronal density and an unchanged proportion of nNOS^+^ neurons (Figs 4 and 5). Given that the same cocktail of antibiotics was used in both studies, these differences suggest that timing of antibiotic treatment may have completely different impacts on the ENS. This differential response of the ENS might potentially be due to different dysbiotic profiles but, unfortunately, this question cannot currently be addressed since the microbiome was not profiled in the study with juvenile mice^18^. All these observations call for further work in this regard, and suggest that analyses of the impact of antibiotic treatment on ENS structure and function should systematically be done in conjunction with an analysis of the gut microbiota.

HAEC is a severe pre- and/or post-operative life-threatening complication of HSCR, still responsible for a high number of HSCR-associated deaths^37,38^. HAEC must be treated with broad-spectrum antibiotics and usage of a cocktail similar to what we used is not rare^37^. The possibility that HAEC is a major cause of death in the *Ednrb*-null HSCR mouse model has been tested using an antibiotic treatment very similar to ours in terms of both timing (starting at pre-natal stages) and composition (cocktail of broad-spectrum antibiotics)^39^. Interestingly, although this treatment did not prevent death of these animals, it did delay the average age of death by several days as we observed for *Tash*T^Tg/Tg^ male pups (Fig. 3A). As our data strongly suggest that this temporal shift may be explained by dysbiosis-induced changes to the structure and composition of the ENS, it would be especially important to analyze the ENS and microbiota of *Ednrb*-null mice exposed to antibiotics. Since one of the impacts that we observed is the worsening of genetically-induced ENS defects, similar studies should be done on as much HSCR mouse models as possible in order to define global and/or specific rules. Such knowledge could in turn be used to eventually adapt current antibiotic treatments as a function of their impact on the microbiota and the ENS in different genetic contexts.

Standard treatment of HSCR involves the resection of the aganglionic segment and reconnection of the most distal innervated region to the anus. Long term motility problems are often present after this surgery^40^, and characterization of different HSCR mouse models strongly suggests that this is due to the structure and/or composition of the most distal ENS^11–15^. The current study now suggests that, when distal aganglionosis is not extensive, this distal ENS might also play a key role in HSCR pathogenesis *per se*. Indeed, our analysis of megacolon-suffering *TashT*^Tg/Tg^ male mice notably revealed an important contribution for increased NO signaling, as highlighted by the fact that L-NAME treatments can promote their survival (Fig. 8C). Since the proportion of nitrergic neurons in these mice can be modulated by antibiotics-induced dysbiosis in a temporal manner (Figs 4 and 5), our work calls for detailed characterization of the distal ENS as a function of age and exposure to antibiotics not only in HSCR mouse models but also in human HSCR patients. Indeed, given that the proportion of nitrergic neurons is increased in HSCR patients as well^41^, this could have a direct influence on how these patients are being taken care of.

## Materials and Methods

### Animals

All experiments were performed according to the guidelines of the Canadian Council on Animal Care (CCAC) and approved by the relevant institutional committee (*Comité institutionnel de protection des animaux*; CIPA reference #899) of University of Quebec at Montreal (UQAM). Euthanasia was performed via CO_2_ inhalation following isoflurane anesthesia. All mice used in this study, including *TashT*^Tg/Tg^ ^15,26^ and *Holstein*^Tg/Tg^ ^30^ mutants, were of the same genetic background (FVB/N). These mice were housed in the same room of UQAM’s animal facility under a 12 h/12 h light/dark cycle, with *ad libitum* access to water and standard chow pellets. Breeding couples were separated upon detection of a vaginal plug, and pups were left with their mothers until weaning at P28. Weaned siblings of the same sex were then housed at 3 to 4 per cage.

For antibiotic treatment, 10-day pregnant mice were exposed to a previously described cocktail of broad-spectrum antibiotics^24^ sweetened with sucrose (or sucrose alone as control) in their drinking water until weaning of their pups. The same procedure was used for newly weaned pups until reaching P36. The cocktail included 0.5 mg.ml^−1^ Vancomycin hydrochloride (V200-25, GoldBio), 0.5 mg.ml^−1^ Neomycin Trisulfate (400–150 XG, Multicell), 1 mg.ml^−1^ Ampicillin Sodium Salt (AB0028, BioBasic), 1 mg.ml^−1^ Metronidazole (M3761-25G, Sigma) and 10 mg.ml^−1^ Sucrose (57-50-1, Amresco), and was refreshed every 48 hours. Bodyweight of control and antibiotic-treated pups was assessed in the morning at P1, P7, P14, P21 and P28, using a high precision digital scale (Kilotech KHA5001).

To locally reduced NO signaling in the colon of antibiotics-treated or megacolon-suffering *TashT*^Tg/Tg^ male pups, 200 µl rectal enemas containing 2 µg.µl^−1^ L-NAME (NG-nitro-L-arginine-methyl ester hydrochloride; N5751-1G, Sigma) or vehicle only (H_2_O) were given once a day during 7 days, starting either at P26 (for antibiotics-treated animals) or upon the detection of symptoms of aganglionic megacolon (growth delay, general weakness and hunched posture)^26^.

### Sampling and quantification of fecal bacteria

To profile the basal microbiome of *TashT*^Tg/Tg^, *Holstein*^Tg/Tg^ and WT mice, the fecal matter of single P21–22 pups (each coming from a different cross) was collected from dissected colons (without the cecum) and immediately transferred into sterile tubes. For all other analyses, mice were individually placed in sterile partitioned cages for at least 1 hour until a sufficient amount of stool was present (between 5–10 fecal pellets). Fecal pellets were then collected for each mouse and pooled into a sterile tube. For P14 pups, feces from up to 3 animals were pooled to obtain enough material for subsequent analysis. Every recovered fecal sample was stored at −80 °C until analysis.

The concentration of bacteria in stools was quantified by slightly modifying a previously described protocol^42^. Briefly, thawed stool samples were weighed and solubilized at a concentration of 10 mg/ml in counting buffer (10 mg.ml^−1^ bovine serum albumin and 0.2 g.l^−1^ NaN_3_ in PBS). After overnight incubation at 4 °C, samples were homogenized and transferred on ice for 1 hour to allow sedimentation of large fiber pieces. A 1:400 dilution of the supernatant was then prepared in counting buffer and filtered through a 40 µm cell strainer (Fisherbrand, cat. No. 22363547). Bacteria were fluorescently labeled by adding SYTO-13 (Molecular Probes, cat. No. S7575) at 5 µM final concentration and, after 10 min of incubation in the dark at room temperature, an additional 1:100 dilution was made in sterile water before analysis on a BD Accuri C6 cytometer (Becton Dickinson Canada, Mississauga, ON) to count the number of bacteria within a 5 min timeframe. The concentration of bacteria was then calculated as a function of final volume and dilution factor, and expressed in bacteria per µl of stool homogenate.

### Amplicon sequencing and bioinformatics

Extraction of bacterial DNA was performed using the QIAamp® Fast DNA Stool Mini Kit (QIAGEN Cat. No. 51604). A total of 26 (2 Forward and 24 Reverse) barcoded primers^43,44^ were then used to amplify the V5-V6 region of the *16S* rRNA gene using the Feldan PCR kit (Bio Basic Inc) and 100 ng of DNA per sample (final volume of 25 µl). PCR conditions consisted of an initial denaturation step of 2 min at 95 °C, followed by 35 cycles of 30 s at 95 °C, 30 s at 64 °C and 30 s at 72 °C, and completed by a final extension of 5 minutes at 72 °C. PCR reactions also included two negative controls: a water blank with a Forward/Reverse primer pair, and 100 ng of DNA from sample number 1 with only a Forward primer (Table S1). To confirm the efficiency of PCR reactions, 5 µl of each sample was visualized on a 2% agarose gel. DNA content from the remaining 20 µl was normalized using the Invitrogen Sequalprep PCR Cleanup and Normalization Kit, and pooled at equal concentrations (0.8 ng/µl). Sequencing was performed on an Illumina MiSeq sequencer as previously described^43,44^.

Raw sequences were paired and processed using the MOTHUR pipeline^45^. Sequences with low quality scores (<30), long homopolymers, ambiguous bases and abnormal length were removed from the dataset. The UCHIME algorithm^46^ was used to remove chimeras. Sequences within a 97% identity threshold were binned into Operational Taxonomic Units (OTUs) and OTUs with a single read (singletons) were removed. Taxonomic classification of the sequences was performed with the BLAST algorithm^47^ and the Greengenes database^48^ implemented in the MOTHUR pipeline. Biom files consisting of OTUs abundance tables and metadata^49^ were generated, and the BIOM package^49^ was then used to import biom files into R^50^ for subsequently exporting taxa abundance tables in CSV format. Piecharts to display the distribution per phyla or order were prepared with Microsoft Excel while all other graphs were prepared with R.

### Tissue preparation and immunofluorescence

Tissue were processed as previously described^15^. Muscle strips (containing the myenteric plexus between both the longitudinal and the circular muscle layers) from the duodenum, ileum and/or colon were either labeled immediately or store at 4 °C in PBS for up to 1 month until subsequent labeling. The position of bowel sub-regions used in this study is indicated where relevant in figures and/or figure legends.

For immunolabeling, muscle strips from relevant bowel segment were incubated 2 hours at room temperature in blocking solution (10% fetal bovine serum, 0.66% Triton-X100 in PBS), and then incubated overnight at 4 °C with different combination of primary antibodies (HuC/D;Calretinin;nNOS, HuC/D;Calretinin, HuC/D;nNOS, HuC/D;ChAT, HuC/D;S100β, Tuj1;S100β or c-Kit;Tuj1). Tissues were subsequently washed in blocking solution and incubated with relevant secondary antibodies for 2 hours at room temperature. All antibodies were diluted in blocking solution. Dilution factors and other antibody details are shown in Table S2. Transverse sections (150 µm) of distal ileum previously doubly labeled with anti-Tuj1 and anti-S100β were prepared using a vibrating blade microtome (Microm HM650V, Thermo Scientific) as previously described^51^.

Confocal images (4 µm-thick stacks) of immunolabeled tissues were captured on a Nikon A1 confocal unit (run with the NIS-Element AR4 software). The number of HuC/D^+^, Calretinin^+^, nNOS^+^, ChAT^+^ and S100β^+^ cells in myenteric ganglia from the duodenum, ileum, proximal-mid colon (MC), proximal-distal colon (DC) and/or mid-DC was counted (using the Image J cell counter manual function) in five random fields of view (0.3923 mm^2^) captured with a 20X objective (Plan Fluor, Multi Immersion, NA 0.75, Nikon). A similar approach was used to count interganglionic c-Kit^+^ interstitial cells of Cajal (ICCs) from the proximal-DC in five random fields of view (0.0449 mm^2^) captured with a 60X objective (Plan Apo, Oil, NA 1.4, Nikon). For each bowel segment, the total number of myenteric HuC/D^+^ neurons or c-Kit^+^ ICCs contained in the five fields of view were determined and expressed in cells per mm^2^ of longitudinal surface area. The proportion of Calretinin^+^, nNOS^+^ and ChAT^+^ neurons as well as the HuC/D^+^: S100β^+^ neuron:glia ratio in myenteric ganglia were evaluated by normalizing the total number of Calretinin^+^, nNOS^+^ or ChAT^+^ cells to that of HuC/D^+^ neurons, and the total number of S100β^+^cells to the sum of HuC/D^+^ and S100β^+^ cells, respectively.

### Gastrointestinal (GI) motility assays

GI motility parameters were evaluated using carmine red dye gavage (for total GI transit time and proximal GI transit rate) or the bead expulsion assay (for distal colonic transit time) as previously described^15^.

### Statistical analysis

Data are presented as the mean ± standard deviation with the number (n) of biological replicates included in figures and/or figure legends. Where relevant, *P* values were determined using the One-way analysis of variance (ANOVA) with Tukey’s Multiple comparison of means (95% family-wise confidence level) post hoc test, with the exception of Figs 2A, 6A and S7B (Unpaired two-tailed Student’s *t*-test), Fig. 3A (Mantel-Cox test) and Figs 3C and 8A (Chi-square test), and 0.05 was used as significance cut-off value.

## Supplementary information

Supplementary information

## Data Availability

All materials, data and associated protocols will be made promptly available to readers.
