# Supplementary material for: Gut microbiota-mediated Gene-Environment interaction in the TashT mouse model of Hirschsprung disease
Source: Sci Rep. 2019 Jan 24;9:492. doi: 10.1038/s41598-018-36967-z (PMC6345786; doi:10.1038/s41598-018-36967-z)
Supplement: Supplementary file 1 — Supplementary information [file 41598_2018_36967_MOESM1_ESM.pdf]

Gut microbiota-mediated Gene-Environment interaction in the *TashT* mouse model of Hirschsprung disease

Aboubacrine Mahamane Touré[1,2,3], Mathieu Landry[1,4], Ouliana Souchkova[1,2,4], Steven W. Kembel[1,2] and Nicolas Pilon[1,2 *]

[1] Département des Sciences Biologiques, Université du Québec à Montréal, Montréal H3C 3P8, Québec, Canada.
[2] Centre d'Excellence en Recherche sur les Maladies Orphelines – Fondation Courtois (CERMO-FC), Université du Québec à Montréal, Montréal H2X 3Y7, Québec, Canada.
[3] Département d'Enseignement et de Recherche de Biologie de la Faculté des Sciences et Techniques de l'Université des Sciences, des Techniques et des Technologies de Bamako, Badalabougou, Colline de Badala, Bamako, Mali.
[4] These authors contributed equally

*** Correspondence to**: Nicolas Pilon (pilon.nicolas@uqam.ca)

SUPPLEMENTARY INFORMATION (10 figures and 2 tables):

Fig.S1. Sex-stratified analysis of colonic bacterial composition in wild-type mice at P21-22.

Fig.S2. Genotype- and phenotype-stratified analysis of colonic bacterial composition in wild-type, *TashT*$^{Tg/Tg}$ and *Holstein*$^{Tg/Tg}$ male mice at P21-22.

Fig.S3. Sex- and phenotype-stratified analysis of colonic bacterial composition in *TashT*$^{Tg/Tg}$ mice at P21-22.

Fig.S4. Alpha- and beta-diversity comparisons of gut microbiomes of wild-type and megacolon mouse lines at P21-22.

Fig.S5. Alpha- and beta-diversity comparisons of gut microbiomes of wild-type and *TashT*$^{Tg/Tg}$ P28-30 mice exposed or not to antibiotics.

Fig.S6. Genotype-stratified analysis of colonic bacterial composition as a function of antibiotic treatment in WT and *TashT*$^{Tg/Tg}$ mice at P28-30.

Fig.S7. Impact of antibiotic treatment on bodyweight, cecum size and mucosal glia.

Fig.S8. Representative images of the quantification of neuronal density and proportion of nitrergic and cholinergic neurons.

Fig.S9. Impact of antibiotics-induced dysbiosis on myenteric neuronal density and proportion of nitrergic neurons in the mid-colon of P30-36 wild-type and *TashT*$^{Tg/Tg}$ male mice.

Fig.S10. Proportion of myenteric Calretinin^{+} neurons, neuron:glia ratio and number of interstitial cells of Cajal in the distal colon of P30-36 wild-type and *TashT*$^{Tg/Tg}$ male mice exposed or not to antibiotics.

Table S1. Information on samples used for amplicon sequencing analysis of the gut microbiota

Table S2. List of primary and secondary antibodies used in this study

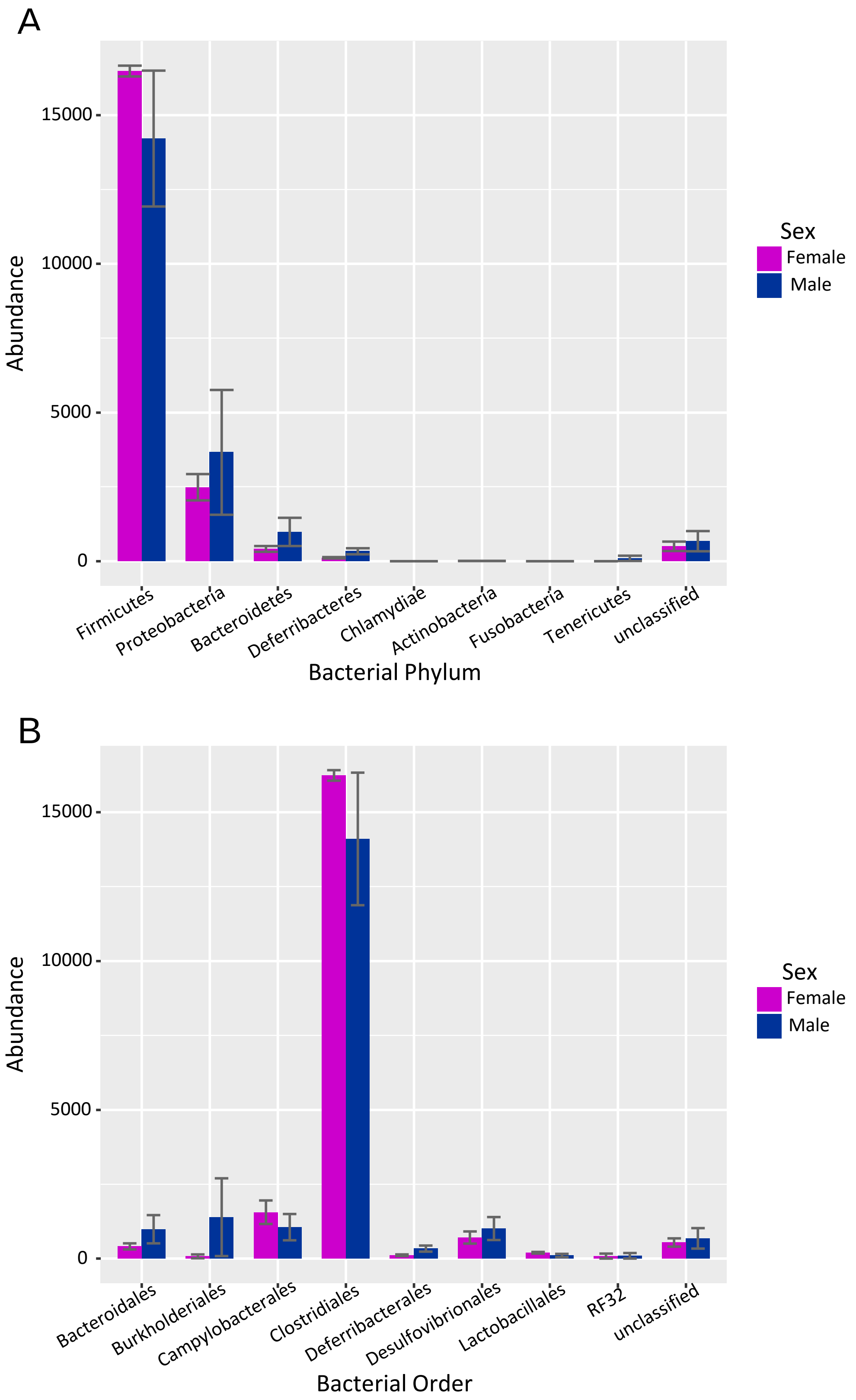

Supplemental Figure 1. Sex-stratified analysis of colonic bacterial composition in wild-type mice at P21-22. (A, B) The bar histograms display the relative average abundance of *16S* rRNA gene sequences at the phylum (A) and order (B) levels. "Unclassified" stands for the sum of yet unknown taxa.

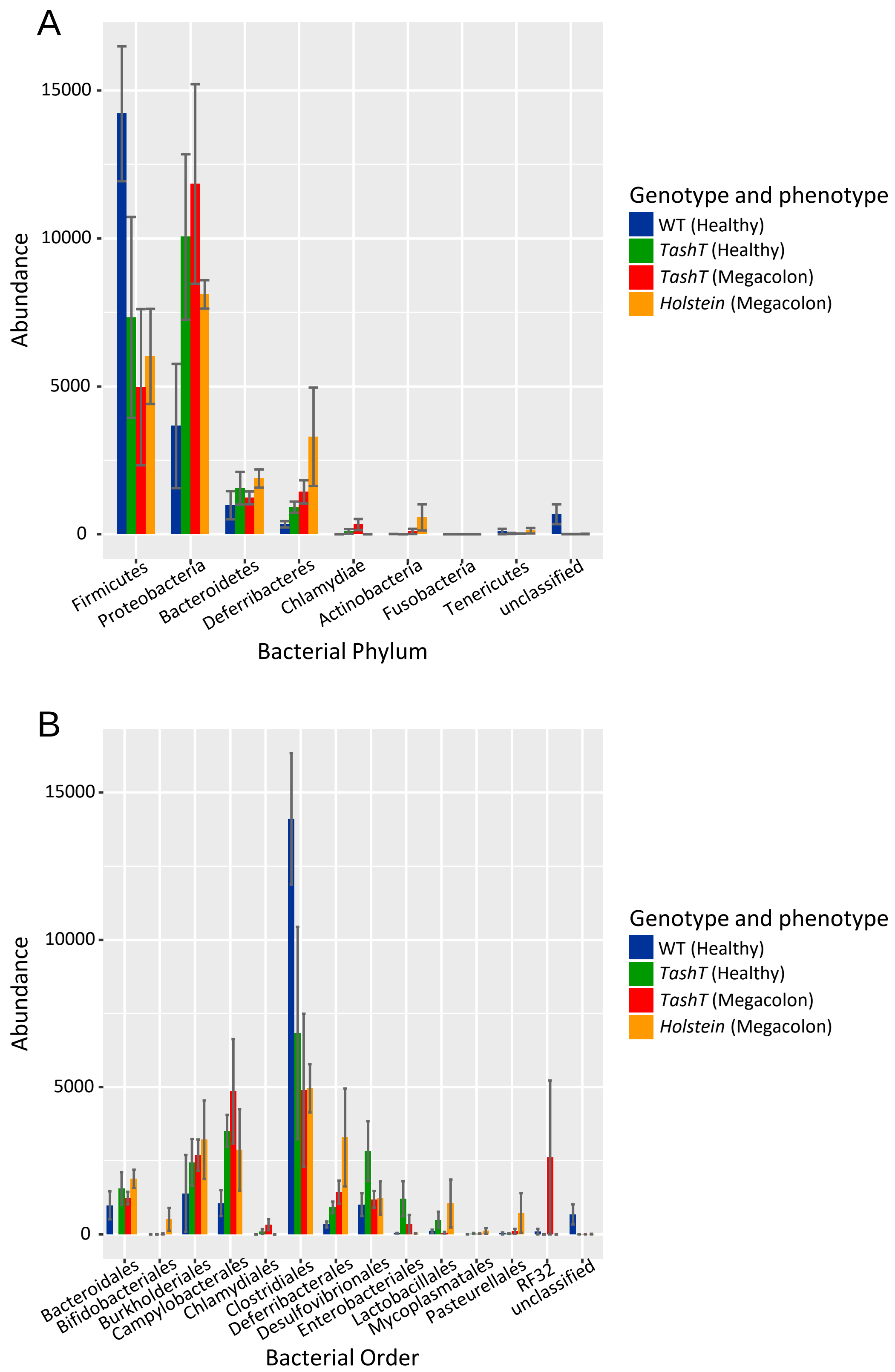

Supplemental Figure 2. Genotype- and phenotype-stratified analysis of colonic bacterial composition in wild-type, *TashT*$^{Tg/Tg}$ and *Holstein*$^{Tg/Tg}$ male mice at P21-22. (A, B) The bar histograms display the relative average abundance of *16S* rRNA gene sequences at the phylum (A) and order (B) levels. "Unclassified" stands for the sum of yet unknown taxa.

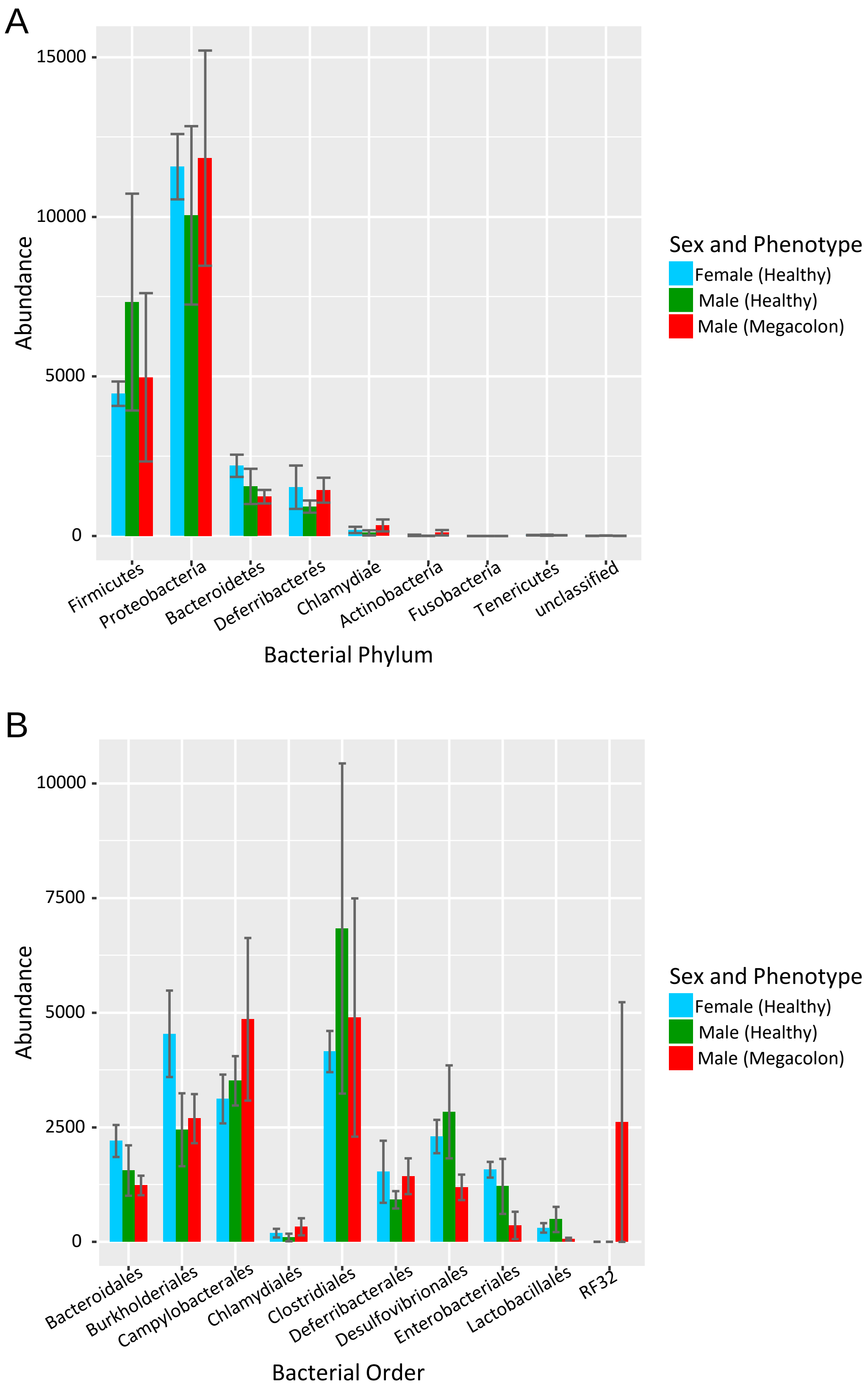

Supplemental Figure 3. Sex- and phenotype-stratified analysis of colonic bacterial composition in *TashT*$^{Tg/Tg}$ mice at P21-22. (A, B) The bar histograms display the relative average abundance of *16S* rRNA gene sequences at the phylum (A) and order (B) levels. "Unclassified" stands for the sum of yet unknown taxa.

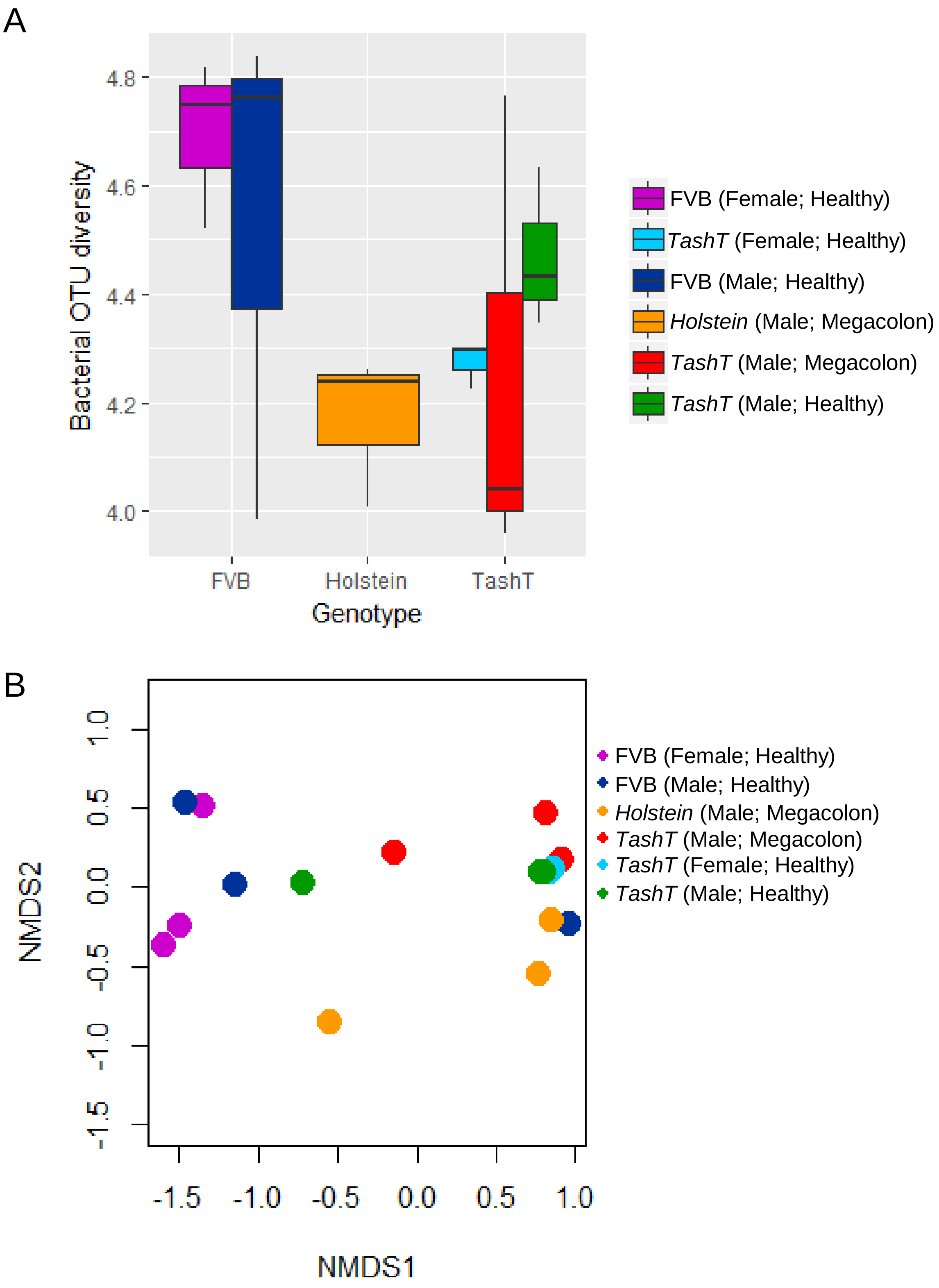

Supplemental Figure 4. Alpha- and beta-diversity comparisons of gut microbiomes of wild-type and megacolon mouse lines at P21-22. (A) Alpha diversity based on Shannon diversity of OTU relative abundances. **(B)** Beta diversity based on non-metric multidimensional scaling (NMDS) of Bray-Curtis dissimilarity of OTU relative abundances among samples.

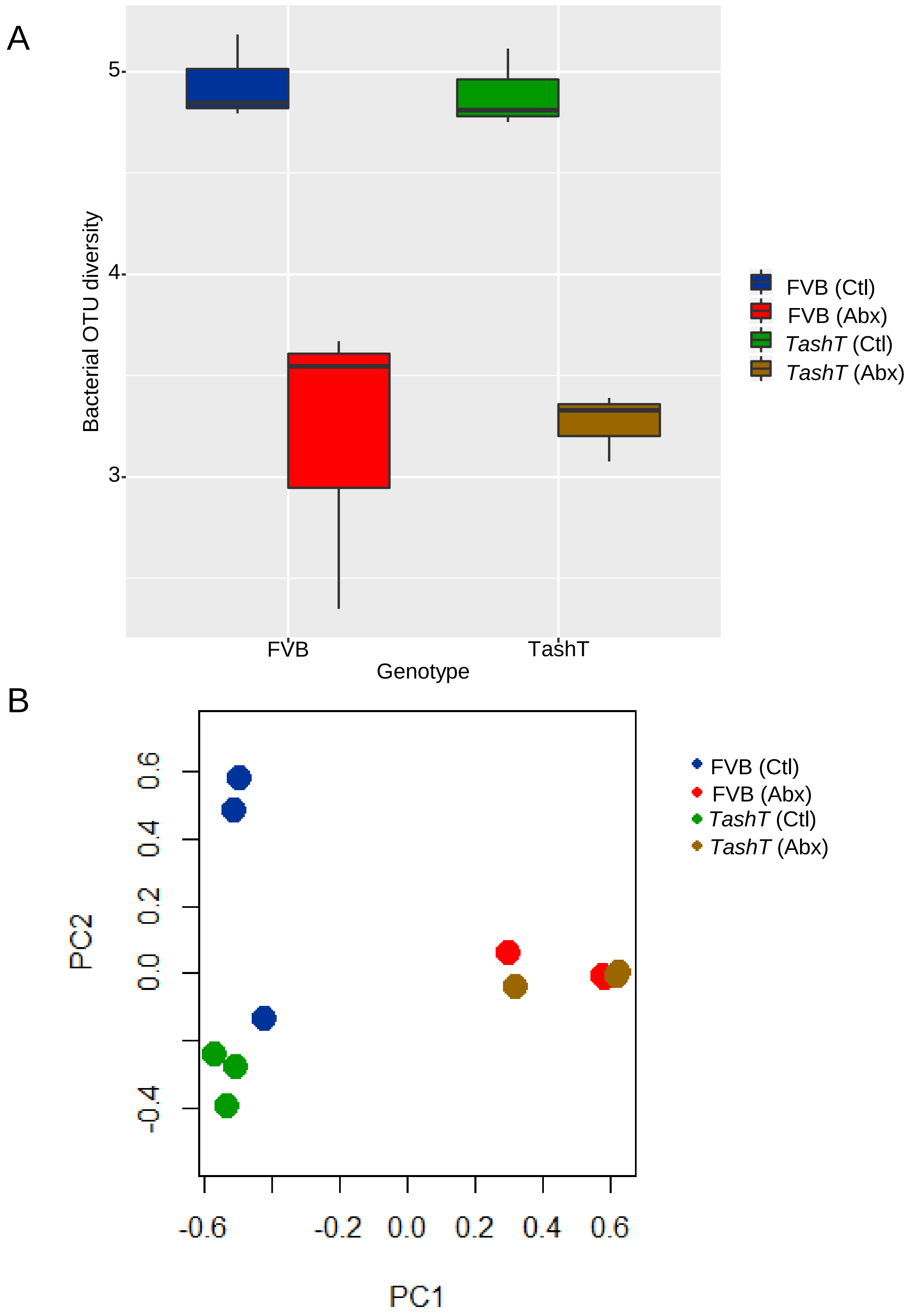

Supplemental Figure 5. Alpha- and beta-diversity comparisons of gut microbiomes of wild-type and *TashT*$^{Tg/Tg}$ P28-30 mice exposed or not to antibiotics. **(A)** Alpha diversity based on Shannon diversity of OTU relative abundances. **(B)** Beta diversity based on principal components (PC) analysis of Hellinger transformed OTU relative abundances among samples.

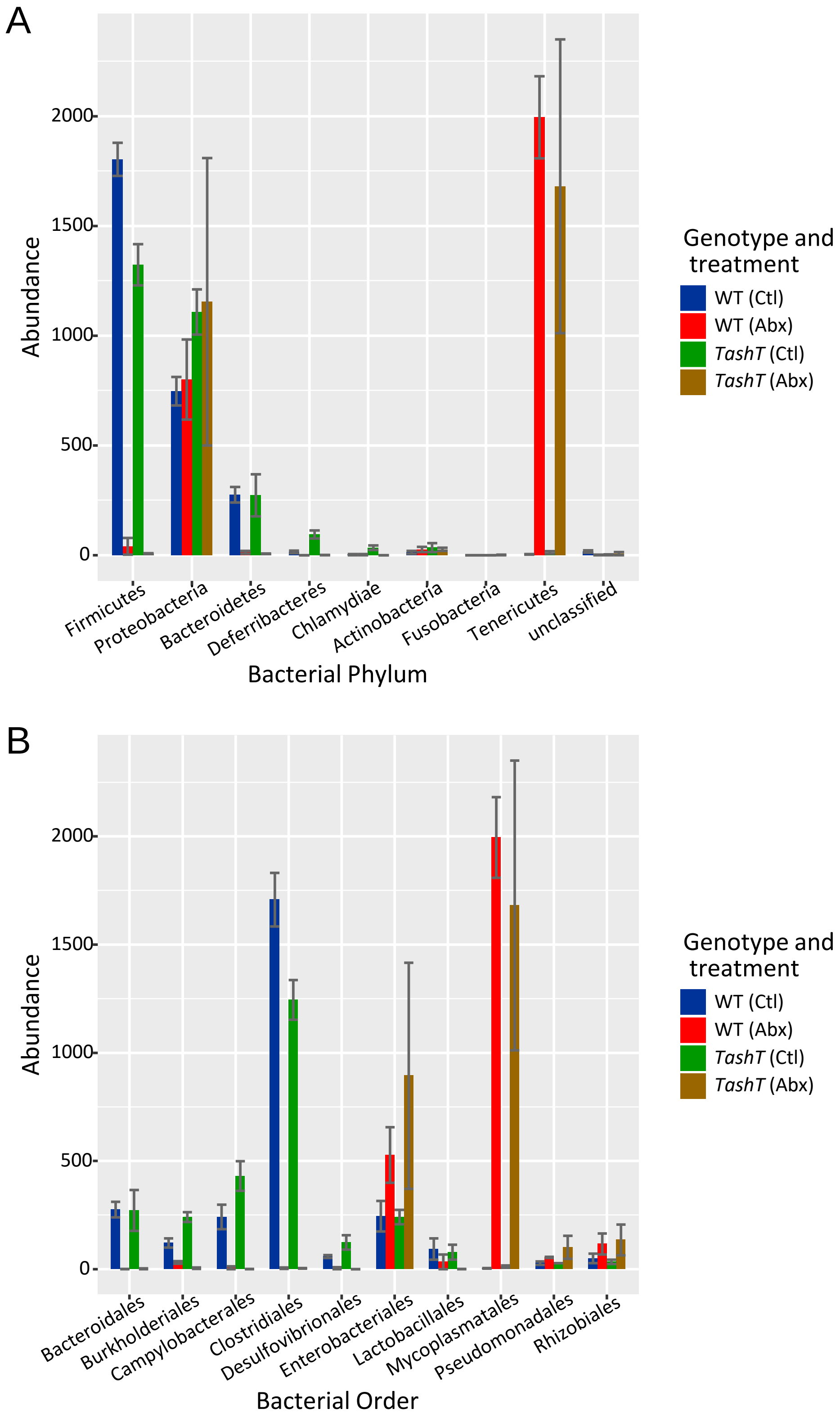

Supplemental Figure 6. Genotype-stratified analysis of colonic bacterial composition as a function of antibiotic treatment in WT and *TashT*$^{Tg/Tg}$ mice at P28-30. (A, B) The bar histograms display the relative average abundance of *16S* rRNA gene sequences at the phylum (A) and order (B) levels. “Unclassified” stands for the sum of yet unknown taxa.

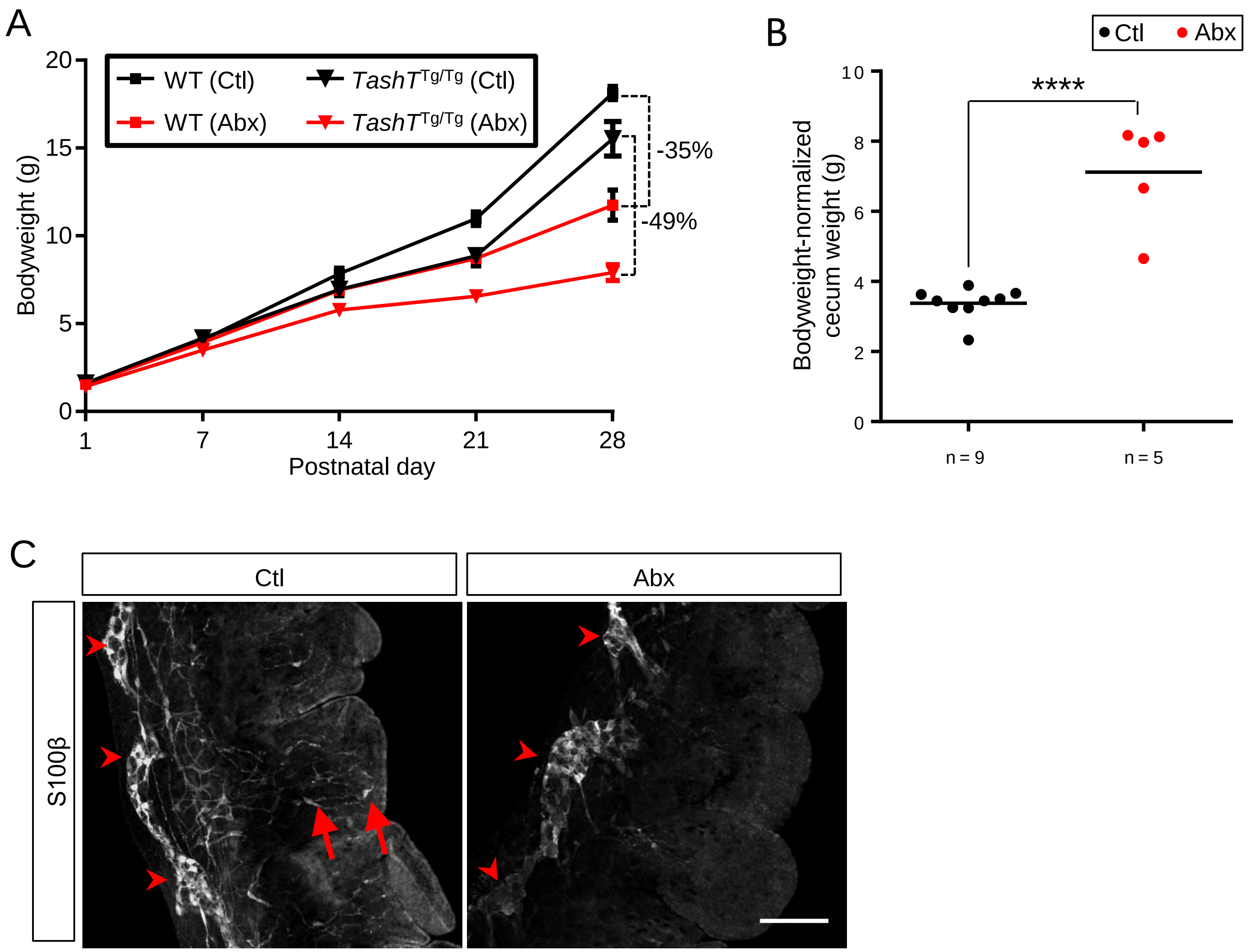

Supplemental Figure 7. Impact of antibiotic treatment on bodyweight, cecum size and mucosal glia. **(A-B)** Exposure to antibiotics causes progressive growth retardation (A) as well as an enlarged cecum (B; shown here is data for P30-36 animals) in both wild-type and *TashT*$^{Tg/Tg}$ mice**. (C)** Representative confocal images of enteric glial cells labeled with anti-S100β showing the specific depletion of mucosal glia (arrows) in the distal ileum of antibiotics-treated P30-36 mice, while myenteric glia (arrowheads) are not affected. Scale bar, 100µm. (**** $P < 0.0001$; Student's *t*-test)

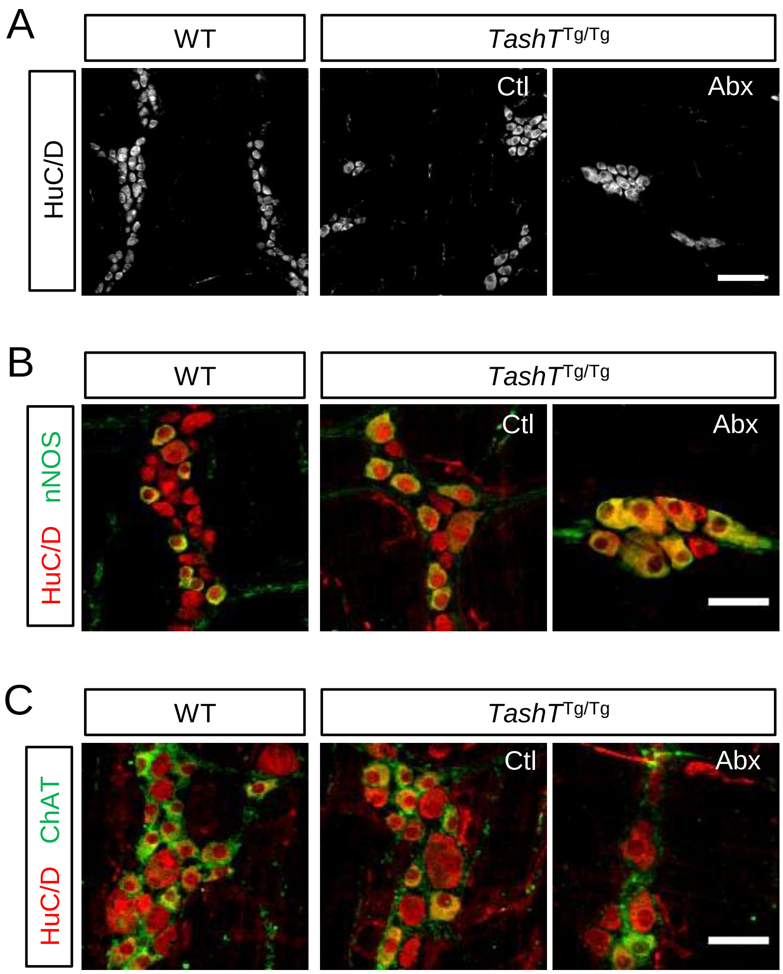

Supplemental Figure 8. Representative images of the quantification of neuronal density and proportion of nitrergic and cholinergic neurons. (A-C) Confocal images of myenteric neurons labeled with anti-HuC/D only (A), or in combination with anti-nNOS (B) or anti-ChAT (C), in the distal colon of P30-36 WT or *TashT*$^{Tg/Tg}$ males exposed (Abx) or not (Ctl) to antibiotics. Scale bar, 100µm (A) and 50µm (B, C).

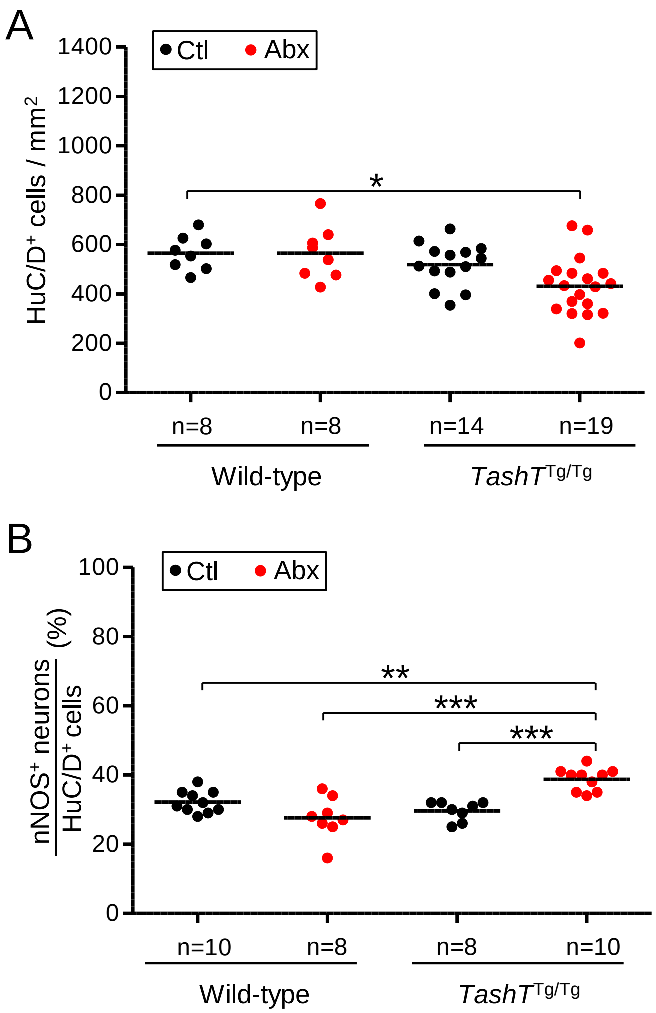

Supplemental Figure 9. Impact of antibiotics-induced dysbiosis on myenteric neuronal density and proportion of nitrergic neurons in the mid-colon of P30-36 wild-type and *TashT*$^{\mathbf{Tg/Tg}}$ male mice. (A) Neuronal density was evaluated via immunofluorescence labeling of the pan-neuronal marker HuC/D and is expressed as the number of neurons per mm^2 of longitudinal surface area. **(B)** The proportion of nitrergic ($nNOS^+$) neurons was evaluated by double-immunofluorescence labeling and is expressed as the percentage of nitrergic neurons among the total number of neurons. (*$p < 0.05$, ** $P<0.01$, ***$p < 0.001$; one-way ANOVA with Tukey's post hoc test)

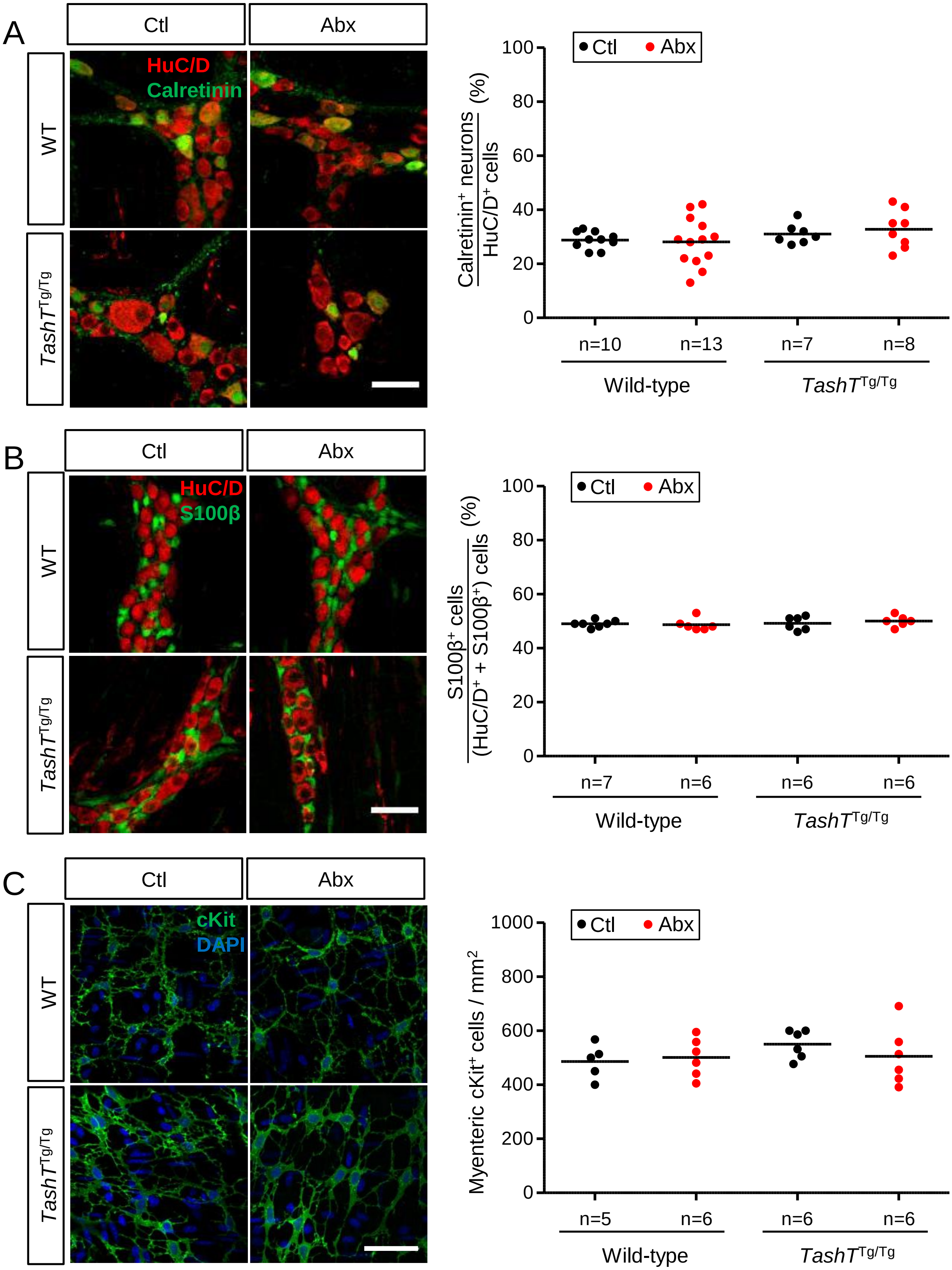

Supplemental Figure 10. Proportion of myenteric Calretinin^{+} neurons, neuron:glia ratio and number of interstitial cells of Cajal in the distal colon of P30-36 wild-type and *TashT*$^{Tg/Tg}$ male mice exposed or not to antibiotics. (A-C) Representative confocal images of myenteric ganglia (A-B) or myenteric interstitial cells of Cajal (C) labeled with the indicated antibodies, and with their associated quantifications shown on the right. The proportion of Calretinin+ neurons (A), neuron:glia ratio (B) and number of interstitial cells of Cajal all remain unaffected by genotype and exposition to antibiotics. Scale bar, 50µm.

Supplemental Table 1. Information on samples used for amplicon sequencing analysis of the gut microbiota

Sample information					Treatment	
Number	Genotype	Gender	Age	Megacolon	Ctl	Abx
1	Wild-type	Male	P28	-	+	-
2	Wild-type	Male	P28	-	+	-
3	Wild-type	Male	P28	-	+	-
4	Wild-type	Male	P28	-	-	+
5	Wild-type	Male	P28	-	-	+
6	Wild-type	Male	P28	-	-	+
7	*TashT*$^{Tg/Tg}$	Male	P28	-	+	-
8	*TashT*$^{Tg/Tg}$	Male	P28	-	+	-
9	*TashT*$^{Tg/Tg}$	Male	P28	-	+	-
10	*TashT*$^{Tg/Tg}$	Male	P30	-	-	+
11	*TashT*$^{Tg/Tg}$	Male	P30	-	-	+
12	*TashT*$^{Tg/Tg}$	Male	P30	-	-	+
13	Wild-type	Male	P22	-	-	-
14	Wild-type	Male	P22	-	-	-
15	Wild-type	Male	P21	-	-	-
16	Wild-type	Female	P22	-	-	-
17	Wild-type	Female	P22	-	-	-
18	Wild-type	Female	P21	-	-	-
19	*TashT*$^{Tg/Tg}$	Male	P22	-	-	-
20	*TashT*$^{Tg/Tg}$	Male	P22	-	-	-
21	*TashT*$^{Tg/Tg}$	Male	P22	-	-	-
22	*TashT*$^{Tg/Tg}$	Female	P22	-	-	-
23	*TashT*$^{Tg/Tg}$	Female	P22	-	-	-
24	*TashT*$^{Tg/Tg}$	Female	P22	-	-	-
25	*TashT*$^{Tg/Tg}$	Male	P22	+	-	-
26	*TashT*$^{Tg/Tg}$	Male	P22	+	-	-
27	*TashT*$^{Tg/Tg}$	Male	P22	+	-	-
28	*Hol*$^{Tg/Tg}$	Male	P22	+	-	-
29	*Hol*$^{Tg/Tg}$	Male	P22	+	-	-
30	*Hol*$^{Tg/Tg}$	Male	P22	+	-	-
Control	Water blank	-	-	-	-	-
Control	sample #1 DNA + F*	-	-	-	-	-

* A forward primer

Supplemental Table 2. List of primary and secondary antibodies used in this study

Antibody	Dilution	Host species	RRID number	Source
Anti-HuC/D	1:500	Mouse	AB_2314656	Molecular Probes, A-21271
Anti-Calretinin	1:500	Goat	AB_10000342	Swant, CG1
Anti-nNOS	1:1000	Rabbit	AB_91824	Millipore, AB5380
Anti-ChAT	1:50	Goat	AB_2079751	Millipore, AB144P
Anti-S100β	1:500	Rabbit	AB_10013383	DakoCytomation, Z0311
Anti-cKit	1:500	Goat	AB_354750	Cederlane (Canada), AF1356
Anti-βIII Tubulin (Tuj1)	1:200	Mouse	AB_2256751	Abcam, ab78078
Anti-Mouse Alexa Fluor 647	1:500	Donkey	AB_2340862	Jackson Immuno Research, 715-605-150
Anti-Rabbit Alexa Fluor 594	1:500	Donkey	AB_2340621	Jackson Immuno Research, 711-585-152
Anti-Goat Alexa Flour 594	1:500	Bovine	AB_2340884	Jackson Immuno Research, 805-585-180
Anti-Goat Alexa Fluor 488	1:500	Bovine	AB_2340883	Jackson ImmunoResearch, 805-545-180
